# Synthesis, Properties Characterization and Applications of Various Organobismuth Compounds

**DOI:** 10.3390/molecules16054191

**Published:** 2011-05-20

**Authors:** Jingfei Luan, Lingyan Zhang, Zhitian Hu

**Affiliations:** State Key Laboratory of Pollution Control and Resource Reuse, School of the Environment, Nanjing University, Nanjing 210093, China; Email: zhanglingyanyaner@163.com (L.Z.), huzt123@126.com (Z.H.)

**Keywords:** organobismuth compounds, synthesis, properties, reagent, catalyst

## Abstract

Organobismuth chemistry was emphasized in this review article due to the low price, low toxicity and low radioactivity characteristics of bismuth. As an environmentally-friendly class of organometallic compounds, different types of organobismuth compounds have been used in organic synthesis, catalysis, materials, *etc.* The synthesis and property characterization of many organobismuth compounds had been summarized. This review article also presented a survey of various applications of organobismuth compounds in organic transformations, as reagents or catalysts. The reactivity, reaction pathways and mechanisms of reactions with organobismuths were discussed. Less common and limiting aspects of organobismuth compounds were also briefly mentioned.

## 1. Introduction

Organometallic compounds such as organoboron, organotin, organosilicon, organoantimony, organolead, *etc.* have been widely applied in organic synthesis in the past several decades [1,2,3]. A number of reports [4,5,6,7,8] on these compounds have mentioned their positive role in a variety of synthetic transformations or polymerizations, acting as reagents or catalysts. Bismuth is relatively low-cost, easily available, and it presents the lowest toxicity among the heavy non-radioactive main group elements [9,10]. These attractive features, combined with the Green Chemistry principles, have made many inorganic and organic derivatives of bismuth broadly and safely used in organic synthesis, catalysis, materials and even medicine [11,12,13,14,15,16]. Hence, organobismuth compounds have emerged as an important class of organometallic compounds that have seen increased applications in different areas over the last 20 years [8,17], as organobismuth chemistry has developed rapidly and some novel organobismuth compounds have been reported [18,19,20,21]. Many types of organobismuth compounds have been proven to be efficient reagents in *C*-, *N*- and *O*-arylation reactions [9], benzoylation reactions [22], cross-coupling reactions [10], *etc.* However, the organometallic chemistry of bismuth has developed relatively slowly compared to that of other main group metals due to the instability of the bismuth carbon bond [23,24]. Up till now, the exploration and information on the chemistry of organobismuth species has been relatively limited [25,26]. For example, application of organobismuth compounds as reagents to C–C bond formations was relatively scarce [1,10]. The poor *σ*-donor power and the reactive Bi–C bonds which were fissile in the presence of some metals resulted in few studies on bismuthine ligands [27]. Moreover, examples where organobismuth compounds acted as catalysts in organic synthesis were even less numerous [28].

In view of the fact that organobismuth compounds have been attracting growing attention to their utilization in various fields with promising novel reactivities, more work is needed in this field, including the design of new organobismuth compounds. Previous review articles written by Gilman *et al.* [29], Finet *et al.* [30], Suzuki *et al.* [31], Zhang *et al*. [32], Jiang *et al*. [33] were already devoted to the chemistry of organobismuth compounds. Hence, this review article was focused specifically on the chemistry of organobismuth compounds, presenting a summary on the synthesis, structural characterization, catalytic and reaction mechanisms and reaction pathways of new organobismuth compounds. The applications of different types of organobismuth compounds in organic synthesis, catalysis, materials science, *etc.* have also been summed up in this review article.

## 2. Synthesis and Property Characterization of Various Organobismuth Compounds

In the past 20 years, many organobismuth compounds have emerged along with the development of the chemistry of organobismuth species. Traditional and novel organobismuth compounds of different types are listed in Table 1.

**Table 1:** Various types of traditional and novel organobismuth compounds.

Compounds	Characterization Methods	Potential Applications	References
BiAr_3_ and BiAr_3_L_2_	NMR, X-ray diffraction	Reagent	[25]
Bu_4_N[PhBiX_2_Y]	IR, FAB^−^ MS, X-ray crystallography	Lewis acid	[35]
(Biphenyl-2,2’-ylene)phenylbismuth diacetate	NMR	Reagent	[36]
Ladder-type organobismuth compounds	GC-MS, NMR, X-ray crystallography	Lewis acid	[40]
Ar_3_Bi=NCOR	NMR, IR, FABMS, X-ray crystallography	Reagent	[42]
Tris[*ortho*-chloromethylphenyl]bismuthane		Reagent	[43]
Ph_4_BiF	X-ray diffraction	Reagent	[47]
(4-CH_3_C_6_H_4_SO_2_NHCH_2_CO_2_)_2_BiAr_3_	Elemental analysis, IR, NMR, MS	Antitumor activity	[16]
Organobismuth chloride and triphenylgermylpropionate	NMR, IR, elemental analysis	Antiproliferative activity	[15]
Resin-bound triarylbismuthanes	NMR	Reagent	[50]
Cyclopropylbismuth	NMR, IR, MS	Reagent	[52]
Ar_3_Bi(OAc)_2_ and Ar_3_BiCl_2_	NMR	Reagent	[9]
[*^t^*BuN(CH_2_C_6_H_4_)_2_Bi]^+^[B(C_6_F_5_)_4_]^−^	NMR	Catalyst	[28]
New dibismuthanes	NMR, X-ray crystallography	Reagent	[54]
Borate ester coordinated organobismuth	NMR, elemental analysis	Reagent	[55]
[2,6-Mes_2_-4-R-C_6_H_2_BiX_2_]_2_	NMR, IR, ESI-MS, MS		[23]
[S(CH_2_C_6_H_4_)_2_Bi(OH_2_)]^+^[ClO_4_]^−^	NMR, X-ray diffraction	Catalyst	[58]
[S(CH_2_C_6_H_4_)_2_Bi(OH_2_)]^+^[OSO_2_C_8_F_17_]^−^	NMR, X-ray diffraction, TG-DSC analysis, Hammett indicator	Catalyst	[59]
C_6_H_11_N(CH_2_C_6_H_4_)_2_BiBF_4_	X-ray analysis, TG-DSC analysis	Catalyst	[60]
Silyl-substituted bismuth	NMR, X-ray analysis	Reagent	[62]
[Ar^1^Ar^2^Ar^3^Ar^4^Bi^+^][X^−^]	NMR	Reagent	[63]
Water-soluble non-ionic triarylbismuthanes	NMR, IR, elemental analysis	X-ray contrast media	[18]
Organobismuth rings (RBi)_3_ and (RBi)_4_	NMR, X-ray analysis		[19]
[(Me_2_Bi)_3_(Tm *^t^*^Bu^)_2_]^+^[Me_2_BiCl_2_]^−^	X-ray diffraction	Reagent	[65]
[2,6-(Me_2_NCH_2_)_2_C_6_H_3_]BiX_2_	NMR, X-ray diffraction		[26]
Bi^V^R_3_(O_2_CR’)_2_	NMR, X-ray diffraction, elemental analysis	Reagent	[67]

### 2.1. Organobismuth(III) and Organobismuth(V) Complexes

Hassan and co-workers reported their efforts on the synthesis of bismuth complexes containing functional groups in the early exploration period of organobismuth chemistry [25]. Two types of organobismuth complexes, bismuth(III) complexes (BiAr_3_) and bismuth(V) complexes (BiAr_3_L_2_) where Ar was a phenyl derivative containing a donor functional group, were prepared and characterized.

**Scheme 1:**
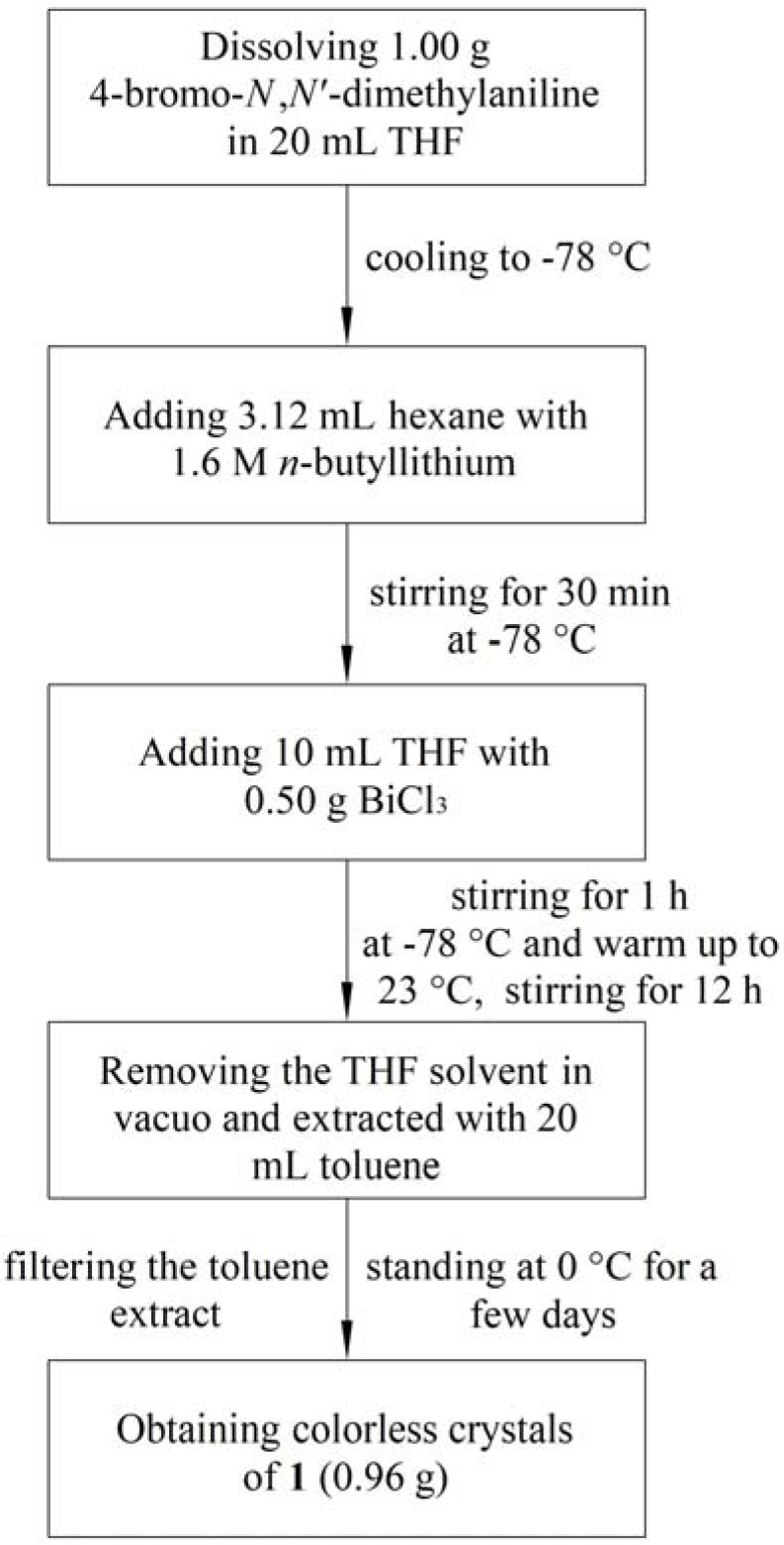
Preparation procedure of Bi[*p*-C_6_H_4_(NMe_2_)]_3_ (**1**) [25].

The model complex Bi[*p*-C_6_H_4_(NMe_2_)]_3_ (**1**) which was previously reported with poor yield by Gilman and Yablunky was newly synthesized by a modified procedure. Taking compound **1** for example, the typical preparation procedure of these compounds is depicted below in Scheme 1. Compound **1** was obtained with a pyramidal geometry which was similar to that of BiPh_3_. Several bismuth(V) derivatives of compounds **1** and Bi{*p*-C_6_H_4_-[CH_2_N(2-Py)_2_]}_3_ (**2**) were synthesized via chlorine oxidation of **1** and **2** and the subsequent substitution reactions. Among above newly synthesized complexes, compound **2** was the first example of a tertiary organobismuth(III) complex containing a chelating functional group while compound Bi{*p*-C_6_H_4_[CH_2_N(2-Py_2_)]}_3_(O_2_CCH_3_)_2_ (**3**) was the first example of a tertiary bismuth(V) complex containing the bidentate dipyridyl functional group.

### 2.2. Mixed Halophenylbismuthates(III)

Mixed halophenylantimonates(III) with Lewis acid character had been successfully prepared by Sharma *et al.* [34], who in the same way synthesized mixed halophenylbismuthates(III) [35]. The formulae of these complexes could be written as Bu_4_N[PhBiX_2_Y] where X = Cl or Br, Y = Cl, Br or I, X ≠ Y. The geometry around bismuth in the Bu_4_N[PhBiCl_2_Br] anion (**4**) was square pyramidal and the molecule existed as a dimer. In the structure of compound **4**, the phenyl group was at the axial position and the four halogen atoms formed the basal plane of the square pyramid for [PhBiCl_2_Br]^−^.

The complexes were prepared by cocrystallization of 1:1 mixtures of Bu_4_NY and PhBiX_2_ from anhydrous methanol where X ≠ Y. The crystals of compound **4** were obtained by solvent diffusion from 1:2 CH_2_Cl_2_-ethanol mixtures at −5 °C over a period of two days while the crystals of Bu_4_N[PhBiBr_2_I] (**5**) were obtained from slow evaporation of its dichloromethane solution.

### 2.3. Pentavalent Biphenyl-2,2’-ylenebismuth Derivatives

**Scheme 2:**
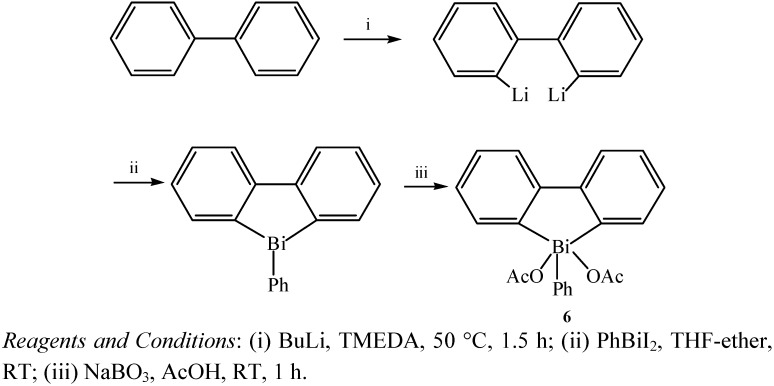
Reactions and conditions for the synthesis of phenylbiphenyl-2,2’-ylenebismuth diacetates (**6**) [36].

Pentacoordinate biphenylylbismuth derivatives, especially the diacetate, which could perform various types of aryl transfer reactions, were investigated by the Fedorov group [36]. The target compound which was identified as (biphenyl-2,2’-ylene)phenylbismuth diacetate (**6)** was obtained under mild conditions with a simple treatment of diacetate (Scheme 2) by a modification of the method of Wittig and Hellwinkel [37].

The (relatively stable) structure including two aryl groups in a heterocyclic bismuth substructure was prepared based on the concept of ligand coupling in heteroaromatic compounds. Structures of this type might enhance the reactivity of biphenylylbismuth derivatives in reactions with different nucleophiles under different conditions. It was reported that the nature of the two extra ligands in pentavalent triarylbismuth derivatives determined the type of reactivity [38].

**Scheme 3:**
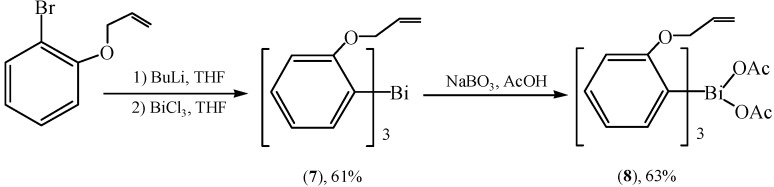
Synthetic route for triarylbismuth derivatives [38].

A similar synthetic route (Scheme 3) had already been applied in the synthesis of triarylbismuth derivatives as early as 1999 [38]. Allylation of the phenolic group, metalation with butyllithium followed by treatment with bismuth chloride afforded the trivalent compound **7**.

Although tris(2-methoxyphenyl)bismuth was oxidized to the corresponding diacetate in relatively good yields by iodobenzene diacetate under neutral conditions, compound **7** was recovered unaffected. Oxidation of the trivalent bismuth compound **7** was accomplished by reaction with sodium perborate in acetic acid, which led to the diacetate **8** in 63% yield. These organobismuths were found to be efficient reagents in the three major types of arylation reactions (*C*-, *N*- and *O*-arylation).

### 2.4. Ladder-Type Organobismuth Compounds

In order to construct new organobismuth compounds by taking the advantage of the combination of multiple inter- and intramolecular Bi-O interactions [39], a novel structure which presented a ladder-type was achieved by Uchiyama and co-workers [40]. Three 1,3,2,4-dioxadibismetane rings successfully comprised this special framework by both inter- and intramolecular Bi–O interactions.

**Scheme 4:**
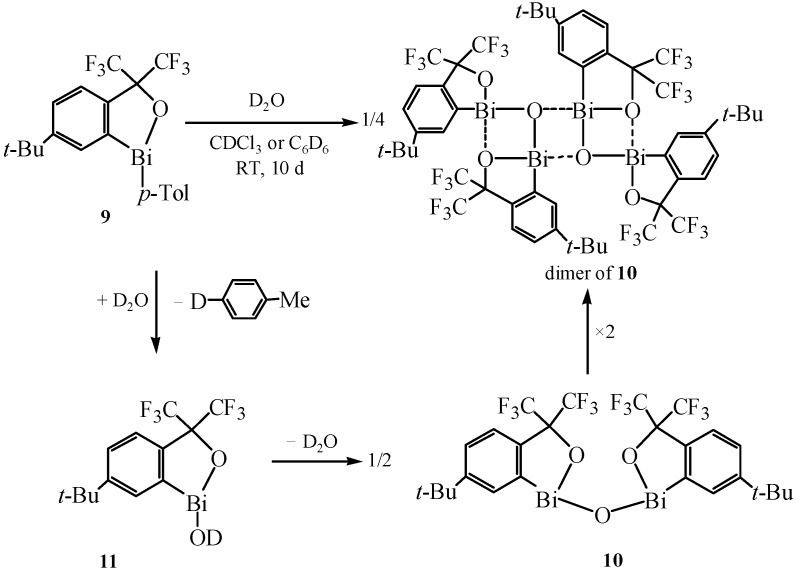
Synthetic route for the ladder-type organobismuth compounds [40].

These novel compounds were prepared by the procedure shown in Scheme 4. By using a method similar to the synthesis of benzoxabismole [41], the benzoxabismole R_f_Bi(C_6_H_4_-4-CH_3_) (R_f_ = –C_6_H_3_-5-C(CH_3_)_3_-2-C(CF_3_)_2_O–) (**9**) with the Martin ligand was prepared first. After that, the dibismuth oxide (R_f_Bi)_2_O **10** was obtained from **9** on standing in CDCl_3_-D_2_O or C_6_D_6_-D_2_O in an NMR tube. By analysis of the GC-MS and ^1^H-NMR spectra of the reaction mixture, researchers concluded that hydrolysis of 6-*tert*-butyl-1-*p*-tolylbenzoxabismole (**9**) followed by self-condensation of hydroxybismuthine **11** resulted in the corresponding dibismuth oxide **10**.

In Scheme 4, it could be seen that two independent molecules of **10** constituted a dimeric structure, and in each independent molecule of **10**, one oxygen atom of the Martin ligand coordinated to the bismuth atom intramolecularly to form Bi–O bonds. X-ray crystallographic analysis showed that the Bi–O intramolecular distances were somewhat longer than the Bi–O intermolecular distances. The strong Bi–O intramolecular coordination was the first example of such intramolecular coordination of the oxygen atom of the Martin ligand. These intramolecular Bi–O interactions constructed two 1,3,2,4-dioxadibismetane rings on both sides of the four-membered ring, eventually providing a ladder type framework.

### 2.5. (Acylimino)triaryl-λ^5^-bismuthanes

**Scheme 5:**
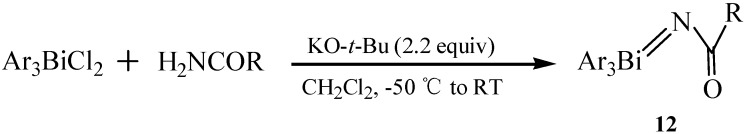
Synthesis of (acylimino)triaryl-λ^5^-bismuthanes [42].

The chemistry of iminopnictoranes had attracted much attention because of their utility in forming bonds to nitrogen in both organic and inorganic reactions. Suzuki *et al.* first synthesized (acylimino)triaryl-λ^5^-bismuthanes of the type Ar_3_Bi = NCOR so as to elucidate the intrinsic properties of the Bi = N bond [42]. As shown in Scheme 5, treatment of *ortho*-substituted triarylbismuth dichlorides (Ar_3_BiCl_2_; Ar = 2-MeC_6_H_4_, 2-MeOC_6_H_4_, 2,4,6-Me_3_C_6_H_2_) with amides (H_2_NCOR; R = CF_3_, CCl_3_, 3,5-(CF_3_)_2_C_6_H_3_) in the presence of 2.2 equiv of KO-*t*-Bu in CH_2_Cl_2_ gave the expected compounds **12**.

The structure and reactivity comparisons were made between (acylimino)triaryl-λ^5^-bismuthane **12** and a series of (acylimino)pnictoranes. Structural observation revealed that the Bi=N bond of **12** possessed a highly polarized single-bond character, probably due to the differences in orbital size and electronegativity between the bismuth and nitrogen atoms. Thermal stabilities of **12** relied on the *ortho*-substituted aryl ligands and the electron-withdrawing *N*-substituents which also afforded kinetic stabilization to the reactive Bi=N bond. Chemical behavior investigations indicated that compound **12** possessed remarkable oxidizing and nitrene-transfer abilities and nucleophilicity, showing potential utility in organic synthesis. This might be attributed to the good leaving ability of the bismuthonio group and the highly polarized character of the Bi=N bonding as well.

### 2.6. Tris[*ortho*-chloromethylphenyl]bismuthane

**Scheme 6:**
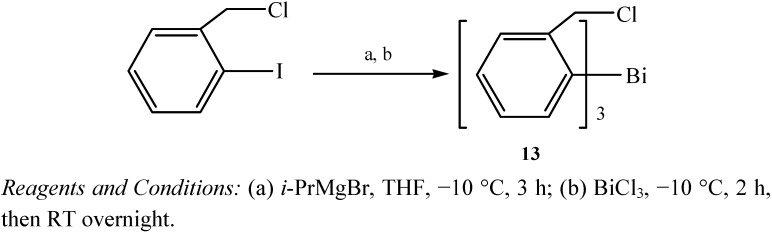
Synthesis of tris[*ortho*-chloromethylphenyl]bismuthane (**13**) [43].

Tris[*ortho*-chloromethylphenyl]bismuthane was synthesized for subsequent three-step one-pot organobismuth-mediated synthesis of benzo[*b*,*d*]pyran compounds [43]. The synthetic procedure of the trivalent organobismuth compound is illustrated in Scheme 6. The reaction of the functionalised aryl-Grignard reagent [44] with bismuth trichloride gave the target compound **13** in moderate yield (27%). Tris[*ortho*-chloromethylphenyl]bismuthane (**13**) was a soft-gray crystalline material which contained two electrophilic centres (–Cl, –Bi).

### 2.7. Fluorotetraphenylbismuth

**Scheme 7:**
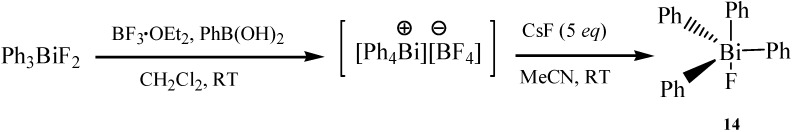
Synthesis of fluorotetraphenylbismuth (**14**) [47].

Tetraphenylbismuth(V) compounds were previously reported to be useful reagents for organic synthesis [45]. However, these compounds were instable because of their weakly coordinating (less nucleophilic) anions. It was reported that fluorine-containing bismuth compounds such as pentafluorophenylbismuth(V) derivatives [46] were strong arylating reagents. It is worth mention that fluorotetraphenylbismuth (**14**) was the first synthesized thermally stable example, taking advantage of its unique amphiphilic property of possessing both nucleophilic (fluorine atom) and electrophilic (phenyl group) moieties on the central bismuth [47]. It was found to be efficient reagent for regioselective α-phenylation. Fluorotetraphenylbismuth (**14**) was obtained through the reactions depicted in Scheme 7.

In the molecular structure of compound **14**, the bismuth center adopts a distorted trigonal bipyramidal geometry with three *ipso* carbons at the equatorial sites and one *ipso* carbon and fluorine atom at the apical sites.

### 2.8. Triarylbismuth(V) Di(N-p-toluenesulfonyl)aminoacetates

Yu *et al.* [16] synthesized a series of triarylbismuth(V) di(*N*-*p*-toluenesulfonyl)aminoacetates to study their cytotoxicity and the influence of amino acid ligands at bismuth on their antitumor activity. Compounds **15**–**18** were synthesized under mild conditions and were formulated as (4-CH_3_C_6_H_4_SO_2_NHCH_2_CO_2_)_2_BiAr_3_ (Ar = Ph, 4-CH_3_C_6_H_4_, 4-ClC_6_H_4_, 4-BrC_6_H_4_). Ar_3_BiCO_3_ (0.5 mmol) was added to a boiling solution of *N*-*p*-toluenesulfonylaminoacetic acid (1 mmol) in acetone (50 mL). Subsequently, the mixture was refluxed for 4 h, cooled and filtered. The starting materials such as *N*-*p*-toluenesulfonylaminoacetic acid and Ar_3_BiCO_3_ were prepared beforehand by the methods reported in the prior literature [48,49]. The solid which was obtained was recrystallized from CH_2_Cl_2_-hexane, to give stable and colorless crystalline solids. The crystal structure of compound **17** showed the bismuth atom existing in distorted trigonal bipyramidal geometry. The bismuth moiety and the nature of the aryl played a positive role on the cytotoxicity of the compounds.

### 2.9. Cyclic Organobismuth(III) Chlorides and their Triphenylgermylpropionate Derivatives

**Scheme 8:**
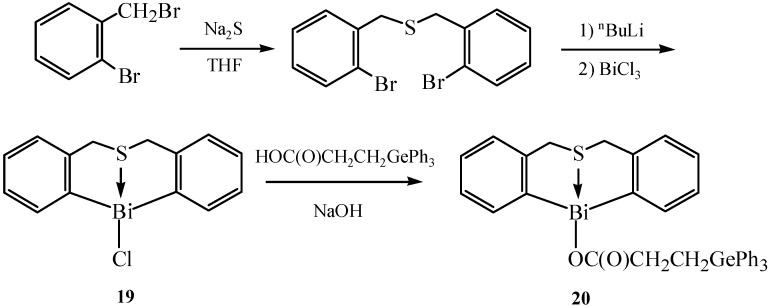
Synthetic route for organobismuth chloride **19** and organobismuth triphenylgermylpropionate **20** [15].

**Scheme 9:**
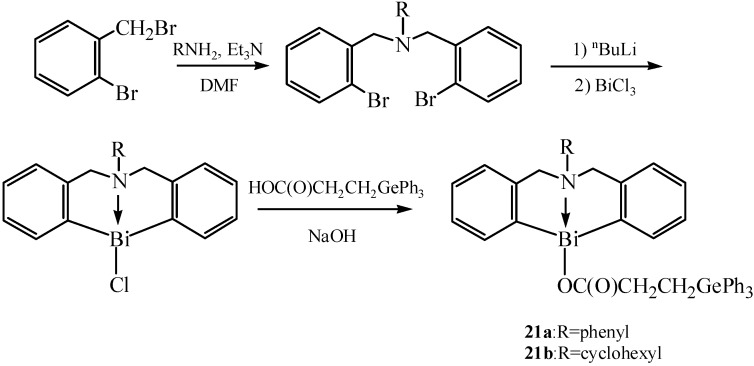
Synthetic route for compounds **21** that bear a nitrogen atom [15].

Zhang *et al.* synthesized six cyclic hypervalent organobismuth (III) chlorides and triphenylgermyl-propionates containing a nitrogen or sulfur atom as intramolecular coordination atom [15]. These cyclic organobismuth compounds showed good antiproliferative activities. The synthetic routes for organobismuth chloride **19** and organobismuth triphenylgermylpropionate **20** are shown in Scheme 8. Compound **20** was obtained through the reaction of **19** with triphenylgermylpropionic acid in the presence of NaOH (as neutralizing agent) and THF-H_2_O (as solvent) under the protection of a N_2_ atmosphere. Scheme 9 presents the synthetic routes for compounds **21** that bear a nitrogen atom.

The eight-membered tetrahydroazabismocine rings were revealed to be highly flexible from the results of single-crystal X-ray analysis. The way the substituted groups acted on the Bi, S or N atom determined the Bi–S or Bi–N bond lengths in the corresponding thiabismocine or azabismocine derivatives. Moreover, the lengthening of Bi–N and Bi–S bond was directly affected by the replacement of Cl atom in azabismocine and thiabismocine with the triphenylgermylpropionic group. The substituents which were connected with the nitrogen atom also had an effect on the Bi-N bond length of azabismocine. Good antiproliferative activities might be related to a suitable coordination ability of Bi^3+^ and the introduction of an organogermanium group.

### 2.10. Resin-Bound Triaryl Bismuthanes

**Figure 1:**
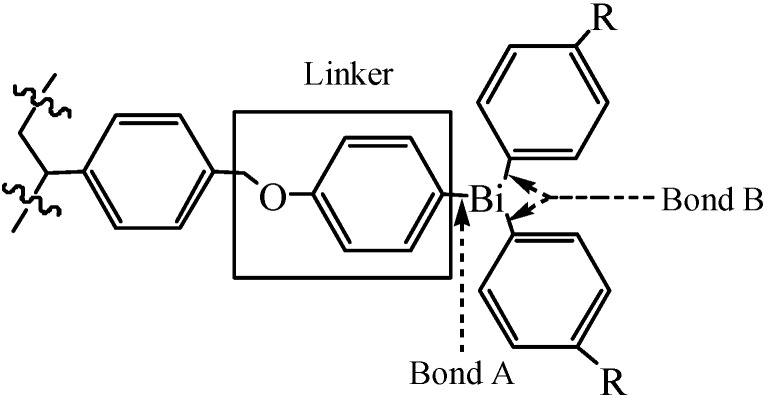
The linker system [50].

Intensive research efforts had been devoted to the study of triorganylbismuth reagents in organic synthesis, and discovery of multidirectional linker strategies for increasing the flexibility in solid-phase organic synthesis (SPOS) had caught the attention of numerous researchers in recent years. Rasmussen and co-workers [50] considered that the combination of the versatile chemistry of triarylbismuthanes coupled with the advantages of solid-phase chemistry would provide a powerful tool for solid-phase and solution-phase synthesis. Thus, they first reported that resin-bound bismuth constituted a novel arylation reagent and its utilization as part of a multidirectional linker system in SPOS. Their method allowed a simple attachment of bismuthanes without polymerization starting from commercially available chloromethylpolystyrene. Furthermore, sufficient discrimination between the two rather similar Bi–sp^2^C bonds A and B which were shown in Figure 1 was achieved. That was a key problem. Cleavage of bond A would result in lower yield and contamination of the products, thus it was crucial to cleave bond B selectively.

**Scheme 10:**
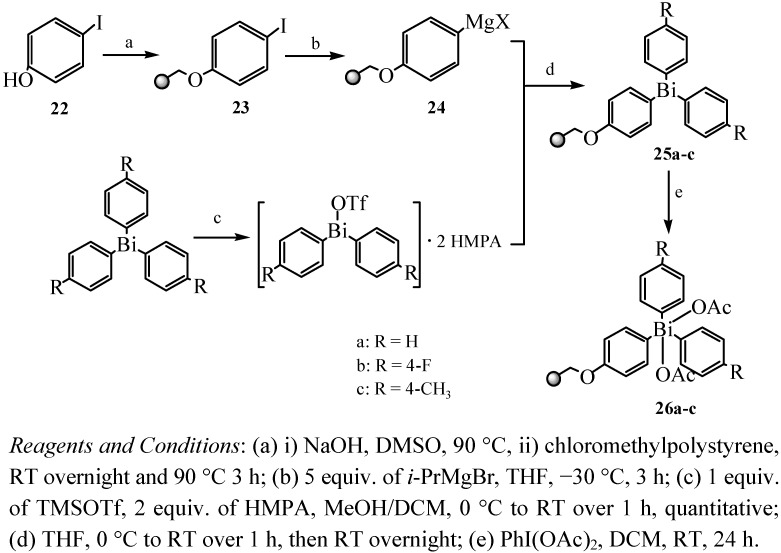
Synthetic route for the resin-bound triarylbismuthanes [50].

Scheme 10 reveals the synthetic route for the resin-bound triarylbismuthanes. Resin **23** was prepared by reaction of 4-iodophenol (**22**) with commercially available chloromethylpolystyrene. In order to improve the required discrimination between the two different types of Bi–sp^2^C bonds in the cleavage step, a phenoxy group was chosen as spacer for the bismuth atom. The resin-bound aryl Grignard compound **24** was prepared from resin **23** by iodomagnesium exchange using isopropyl-magnesium bromide. In order to obtain resin-bound bismuthanes, a suitable bismuth electrophile was needed to be coupled with resin **24**. Ar_2_BiOTf was chosen out of three potential electrophilic bismuth reagents which were BiCl_3_, Ar_2_BiCl, and Ar_2_BiOTf. Three different resin-bound triaryl bismuthanes **25a**–**c** were prepared from resin **24** with loadings from 0.9 to 1.1 mmol/g. Resins **25a**–**c** were subsequently oxidized quantitatively with diacetoxy iodobenzene to the resin-bound triaryl bismuth(V) diacetates **26a**–**c**.

**Scheme 11:**
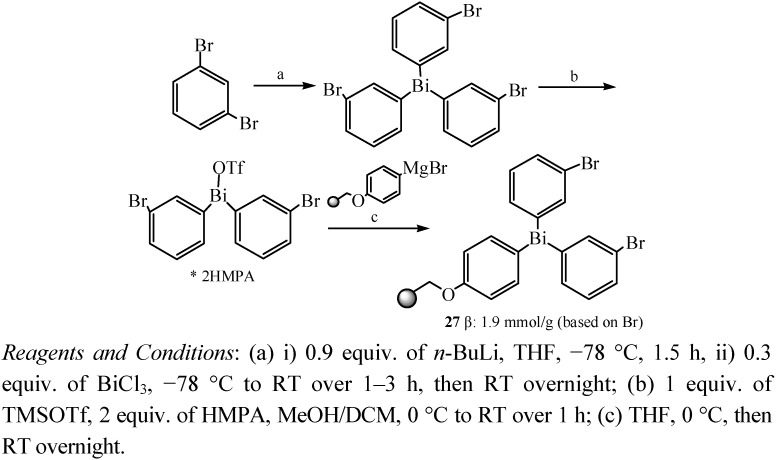
Synthetic route for the resin-bound triarylbismuthanes [51].

Another resin-bound triarylbismuthane **27** was also prepared (Scheme 11) for application in multistep solid-phase organic synthesis [51]. Novel chemoselective cross-coupling reactions with resin-bound triarylbismuthane gave substituted biphenyls in good yield by traceless and multidirectional cleavage of unsymmetrical biphenyls. In addition, both aryl groups of the bismuth linker could be utilized in product formation.

### 2.11. Cyclopropylbismuth

**Scheme 12:**
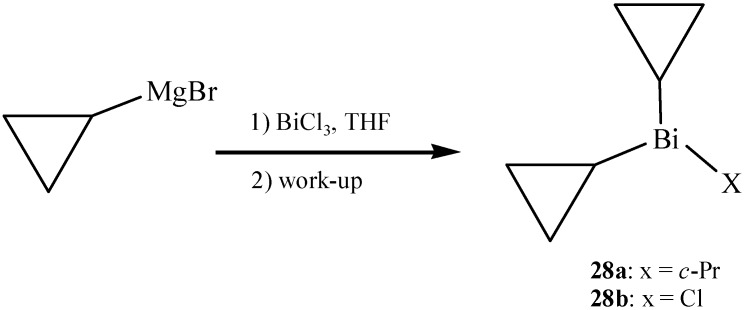
Preparation of cyclopropylbismuth compounds [52].

In consideration of the unique spatial and electronic properties of the cyclopropyl group, Gagnon *et al.* developed a new cyclopropylbismuth reagent which was capable of *N*-cyclopropylating cyclic amides, azoles, and related derivatives [52]. The cyclopropylbismuth compound was prepared in the form of colorless oil, however, compared to other alkylbismuth reagents it was found to be nonpyrophoric. The novel compound, named tricyclopropylbismuth (**28a**) was produced by the addition of cyclopropyl magnesium bromide to bismuth trichloride (Scheme 12). Subsequently the reaction mixture was transferred over brine and the resulted organic layer was evaporated under argon. Mass spectroscopy experiments detected not only **28a**, but also other minor cyclopropylbismuth species such as **28b**.

### 2.12. Tris(polymethoxyphenyl)bismuth Derivatives

Finet *et al.* investigated the synthesis of tris(polymethoxyphenyl)bismuth derivatives and their utilization in *C*-, *N*- and *O*-arylation reactions [9]. These bismuth compounds were prepared with electron-rich aryl groups (polymethoxyaryl fragments) which could be effectively transferred in arylation reactions, breaking the commonly accepted stereotype that organobismuth arylation agents were effective only for the transfer of aryl groups with electron-poor and electron-neutral substituents.

**Scheme 13:**
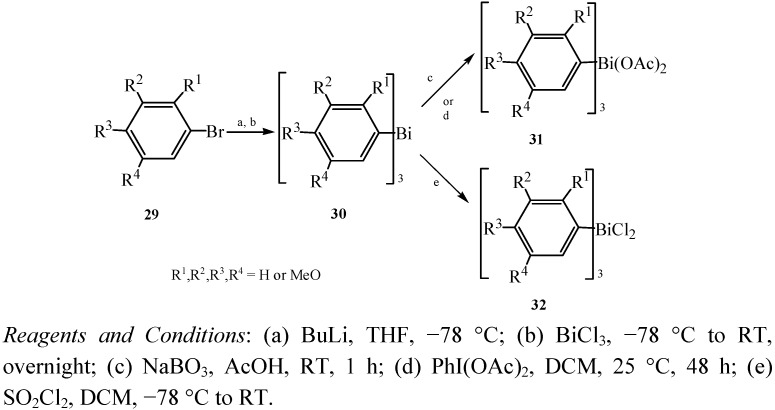
Synthesis of triarylbismuth diacetates **31** and triarylbismuth dichlorides **32** [9].

Synthesis of triarylbismuth diacetates **31** and triarylbismuth dichlorides **32** are shown in Scheme 13. The corresponding triarylbismuthanes **30** were produced by bromine-lithium exchange on compounds **29**, and subsequently a transmetalation reaction with BiCl_3_ followed. Treating the triarylbismuthanes with sodium perborate in acetic acid (c: method A) or by using iodobenzene diacetate as the oxidizing agent (d: method B) gave the triarylbismuth diacetates **31** in relatively good yields. Method A and method B were both utilized in preparing compounds **31**, but method B was not efficient for the oxidation of some of the compounds **31**.

### 2.13. Cationic Organobismuth Complex and Its Coordination Complexes

**Scheme 14:**
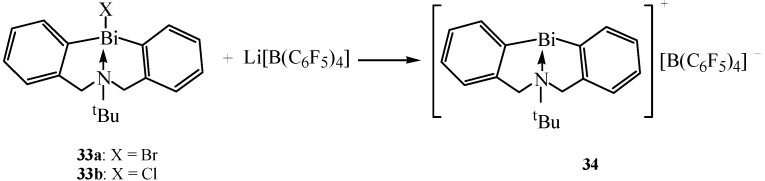
Synthetic route for compound **34 (**[*^t^*BuN(CH_2_C_6_H_4_)_2_Bi]^+^[B(C_6_F_5_)_4_]^−^) [28].

The weak Bi–C bonds of organobismuth compounds cause some instability and consequently limit their application to catalysis. Researchers had found that compounds which possessed stable cyclic frameworks were useful and recoverable reagents for specific organic reactions [53]. Based on these stable cyclic frameworks, Shimada and co-workers continued the research on cationic organobismuth complexes for catalysis [28]. Cationic 5,6,7,12-tetrahydrodibenz[*c*,*f*][1,5]azabismocine with a weakly coordinating borate anion and its coordination complexes with some neutral donor molecules were synthesized. Scheme 14 shows the synthetic route for the representative compound **34 (**[*^t^*BuN(CH_2_C_6_H_4_)_2_Bi]^+^[B(C_6_F_5_)_4_]^−^). As depicted in the Scheme, the reaction of bismuth bromide **33a** or chloride **33b** with Li[B(C_6_F_5_)_4_] gave compound **34** quantitatively.

Under solid state conditions, one of the fluorine atoms of the anion in compound **34** weakly coordinates to the bismuth to form a 10-electron four-coordinate center. In order to study the coordination ability of compound **34**, it was examined with various neutral donor molecules such as aldehydes, methanol, acetonitrile and dichloromethane. Moreover, it was found that the coordination of the donor molecules elongated the coordination distance between the intramolecular nitrogen and bismuth while the degree of elongation depended on their coordination strength. This adjustable property of the intramolecular nitrogen to bismuth coordination stabilizes the cationic complex and is potentially useful for catalysis.

**Scheme 15:**
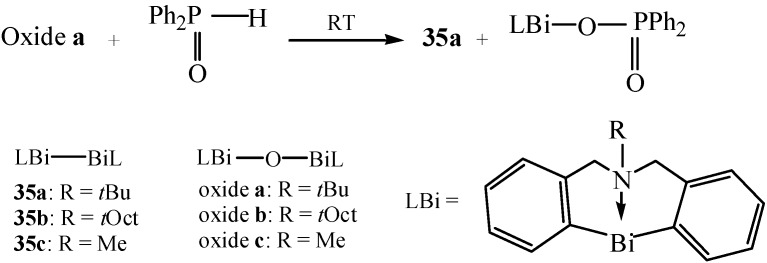
Synthesis of dibismuthanes **35** [54].

Recently, Shimada *et al.* reported a unique reaction for obtaining new dibismuthanes possessing a 5,6,7,12-tetrahydrodibenz[*c*,*f*][1,5]azabismocine framework [54]. The expected dibismuthanes **35** were synthesized by reaction of organobismuth oxides with organophosphorus compounds which possessed a P(=O)H group (Scheme 15). This new procedure was a highly efficient and simple way to prepare **35** in the form of stable crystals. The high reactivity of Bi-Bi bonds which was determined by X-ray analysis in dibismuthanes **35** made them become potentially useful precursors for various bismuth compounds as well as interesting reagents for organic synthesis.

### 2.14. Borate Ester Coordinated Organobismuth Compounds

**Scheme 16:**
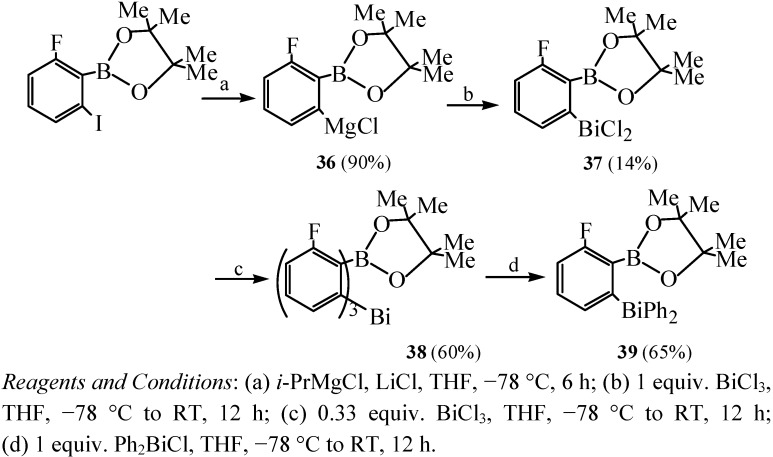
Preparation of borate ester coordinated organobismuth compounds [55].

Caires *et al.* [55] reported the first example of organobismuth compounds which contained organic boron groups. These organobismuth complexes were synthesized with a borate ester moiety because of its relative rarity as an oxygen donor in coordination compounds. These complexes were interestingly probed to have the reactivity of generating arynes at ambient temperature. The above complexes corresponded to the species ArBiCl_2_ (**37**), Ar_3_Bi (**38**), ArPh_2_Bi (**39**), Ar_3_BiCl_2_ (**40**), and ArPh_2_BiCl_2_ (**41**), where Ar = 3-fluoro-2-pinacolatoboronphenyl. As shown in Scheme 16, compounds **37–39** were prepared by reacting Grignard reagent **36** with BiCl_3_ or Ph_2_BiCl. Subsequently, compounds **40** and **41** were prepared by respective treatment of compounds **38** and **39** with an excess of freshly distilled sulfuryl chloride in benzene at ambient temperature. X-ray structures showed that all the complexes **37–41**, except for **40**, gave Bi–O bond distances under 3.0 Å in the solid state, which was between the covalent radii (2.16 Å) and van der Waals radii (3.9 Å). It was consistent with the literature precedent for a Bi–O bonding interaction [56].

### 2.15. Monoorganobismuth(III) Compounds

**Scheme 17:**
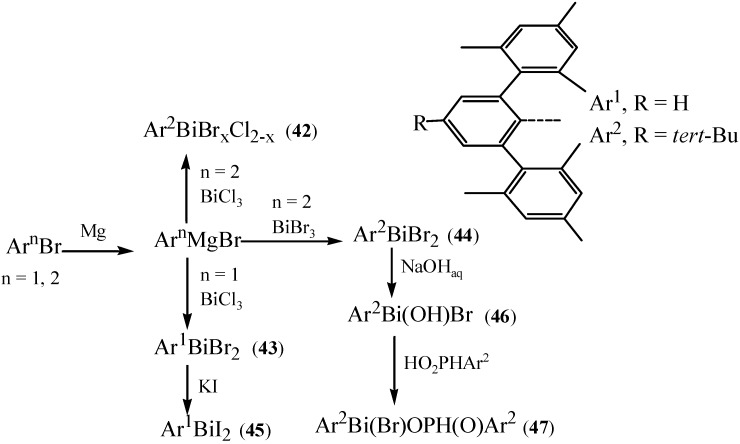
Synthesis of arylbismuth halides **42–47** [23].

Breunig *et al.* synthesized a series of novel sterically congested monoorganobismuth compounds of the following type: [2,6-Mes_2_-4-R-C_6_H_2_BiX_2_]_2_ (**42**, R = *t*-Bu, X = Cl, Br; **43**, R = H, X = Br; **44**, R = *t*-Bu, X = Br; **45**, R = H, X = I) [23]. These special compounds (Scheme 17) had increased stability due to the use of bulky arene ligands. It was reported that arene ligands not only provided steric protection but also stabilized organobismuth derivatives by intramolecular bismuth-arene *π* coordination [57].

By single-crystal X-ray diffraction and additional NMR spectroscopy analysis of its bismuth-arene *π* interactions, compound **47** revealed an unusual 2-fold bismuth-arene coordination. Compound **47** might thus be a suitable model compound for investigating the nature and strength of bismuth-arene *π* complexation.

### 2.16. Air-Stable Organobismuth Compounds

**Scheme 18:**
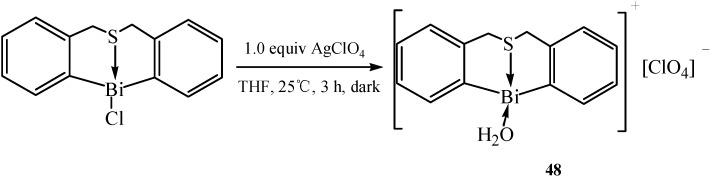
Synthesis of [S(CH_2_C_6_H_4_)_2_Bi(OH_2_)]^+^[ClO_4_]^−^ (**48**) [58].

Most recently, Zhang and co-workers have synthesized a series of air-stable organobismuth compounds which were efficient catalysts for many organic reactions in aqueous media as well as in various organic solvents. The air-stable cationic organobismuth(III) compound [S(CH_2_C_6_H_4_)_2_Bi(OH_2_)]^+^[ClO_4_]^−^ (**48**) [58] was synthesized with both Lewis acidity and basicity. The bismuth center acted as a Lewis acid while the uncoordinated lone electrons of sulfur acted as a Lewis base. The authors claimed that the complex acted as a bifunctional Lewis acid/base catalyst in the direct Mannich reaction. Compound **48** was prepared as shown in Scheme 18.

The molecular structure of compound **48** showed that the oxygen atom of the coordinating water occupied a vacant site of the cationic bismuth center, resulting in a distorted coordination geometry. The perchlorate anion was hydrogen bonded to the coordinated water molecule. The sulfur atom had two lone pairs of electrons: one coordinated with the bismuth center and the other one possibly acting as a Lewis base.

**Scheme 19:**
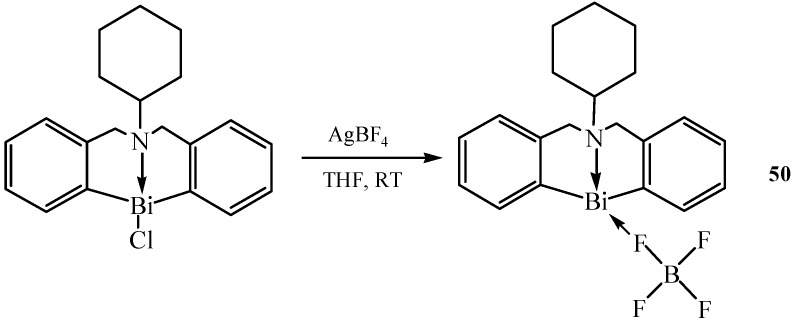
Synthesis of C_6_H_11_N(CH_2_C_6_H_4_)_2_BiBF_4_ (**50**) [60].

Another air-stable cationic compound with the same structure as [S(CH_2_C_6_H_4_)_2_Bi(OH_2_)]^+^[ClO_4_]^−^ which possessed both acidic and basic characters was described in [59]. S(CH_2_C_6_H_4_)_2_BiCl was treated with AgOSO_2_C_8_F_17_ in THF, to afford the expected organobismuth perfluorooctanesulfonate [S(CH_2_C_6_H_4_)_2_Bi(OH_2_)]^+^[OSO_2_C_8_F_17_]^−^ (**49**). This compound was stable up to about 250 °C. Moreover, being air-stable, the compound remained as dry colorless crystals or white powder for more than one year under ambient conditions. Hypervalent organobismuth(III) tetrafluoroborate C_6_H_11_N(CH_2_C_6_H_4_)_2_BiBF_4_ (**50**) [60] was synthesized via a similar synthetic route, as shown in Scheme 19. The central bismuth-containing part of compound **50** showed a pseudotrigonal bipyramidal (TBP) structure.

A strongly electron-withdrawing tetrafluoroborate (BF_4_) was attached to the Bi atom of C_6_H_11_N(CH_2_C_6_H_4_)_2_BiBF_4_, and this compound showed a Lewis acid strength of 3.3 < H_0_ ≤ 4.8 which was stronger than that of C_6_H_11_N(CH_2_C_6_H_4_)_2_Bi(OSO_2_C_8_F_17_) which was previously synthesized [24]. The compound reported in reference [24] was an organobismuth(III) perfluorooctanesulfonate with the formula C_6_H_11_N(CH_2_C_6_H_4_)_2_Bi(OSO_2_C_8_F_17_), showing a weak strength of 4.8 < H_0_ ≤ 6.8.

This might be attributed to the difference between the coordination parts (BF_4_) and (OSO_2_C_8_F_17_). Additionally, C_6_H_11_N(CH_2_C_6_H_4_)_2_BiBF_4_ was highly soluble in methanol and in aqueous solutions of common polar organic molecules. In a test of one year exposure in air, the organobismuth tetrafluoroborate remained as dry crystals and suffered no color change. With all these positive features, C_6_H_11_N(CH_2_C_6_H_4_)_2_BiBF_4_ was evaluated as a Lewis acid catalyst for the allylation of aldehydes and ketones with tetraallyltin.

### 2.17. Silyl-Substituted Bismuth Compounds

**Scheme 20:**
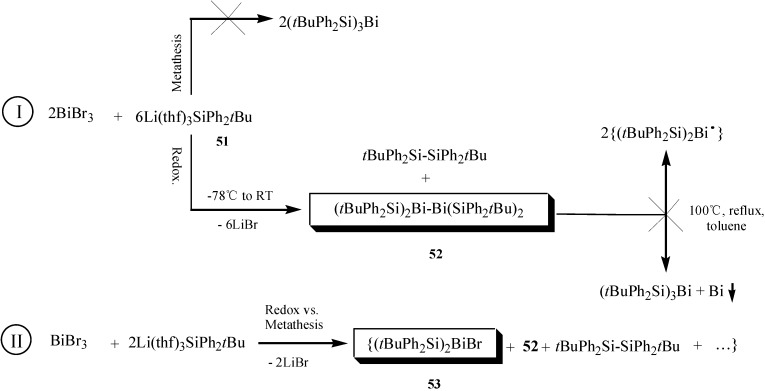
Two reaction pathways for synthesis of bismuthanes **52** and **53** [62].

Since silyl groups could play a stabilizing role in bismuth compounds [61], Monakhov *et al.* synthesized a stable silyl-substituted dibismuthane and a disilylbismuth halide by redox/metathesis reactions of bismuth tribromide (BiBr_3_) with the lithium silanide [Li(thf)_3_SiPh_2_*t*Bu] in various ratios [62]. Stable silyl-substituted dibismuthane and disilylbismuth halides might be potential starting materials in synthetic reactions. As represented in Scheme 20, bismuthanes **52** and **53** were formed via two reaction pathways (I and II).

The reaction pathway II gave compound **53** together with compound **52**. However, main product compound **52** was the sole product in pathway I. Compound **52** displayed high thermostability under reflux at 100 °C for 3 h due to the relatively short Bi–Bi distance. X-ray structure determination showed that in compounds **52** the reactive Bi–Bi bond was effectively surrounded by the bulky *t*BuPh_2_Si groups providing steric protection of the bismuth centers.

### 2.18. Other Special Organobismuth Compounds

**Scheme 21:**
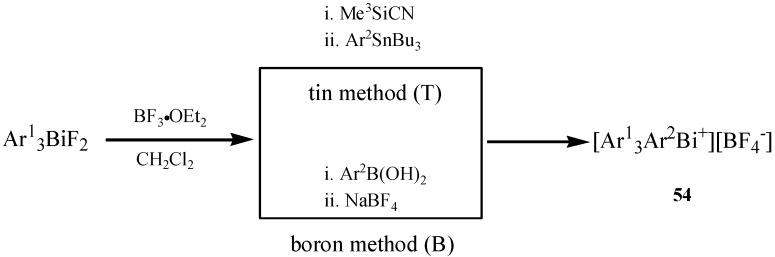
Two methods for synthesis of unsymmetrical tetraarylbismuthonium salts [63].

Unsymmetrical tetraarylbismuthonium salts which possess four different aryl ligands of the type [Ar^1^Ar^2^Ar^3^Ar^4^Bi^+^][X^−^] (**54**) were synthesized *via* two methods involving organotin and organoboron reagents, respectively [63]. The tin method and boron method are shown in Scheme 21. The unsymmetrically substituted tetraarylbismuthonium salts were conveniently produced by the Lewis acid-promoted reaction of triarylbismuth difluorides with arylstannanes or arylboronic acids.

**Scheme 22:**
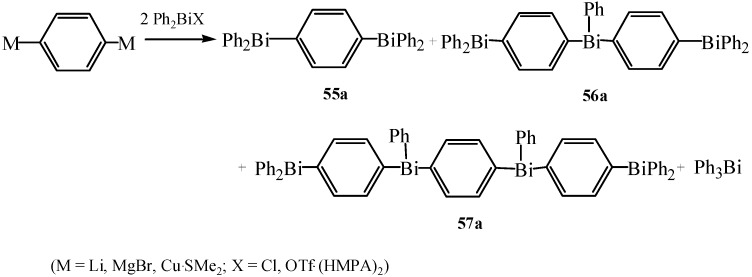
Synthesis of Bi(III)*_n_* and Bi(V)*_n_* compounds [64].

Matano *et al.* synthesized a series of *meta*- and *para*-phenylene-bridged Bi(III)*_n_* and Bi(V)*_n_* compounds (*n* ≥ 2) [64]. The compounds Ph_2_BiC_6_H_4_BiPh_2_ (**55a**), Ph_2_BiC_6_H_4_Bi(Ph)C_6_H_4_BiPh_2_ (**56a**) and Ph_2_BiC_6_H_4_Bi(Ph)C_6_H_4_Bi(Ph)C_6_H_4_BiPh_2_ (**57a**), which bore two or more bismuth atoms, were obtained in 2%–30% yields (Scheme 22).

**Figure 2:**
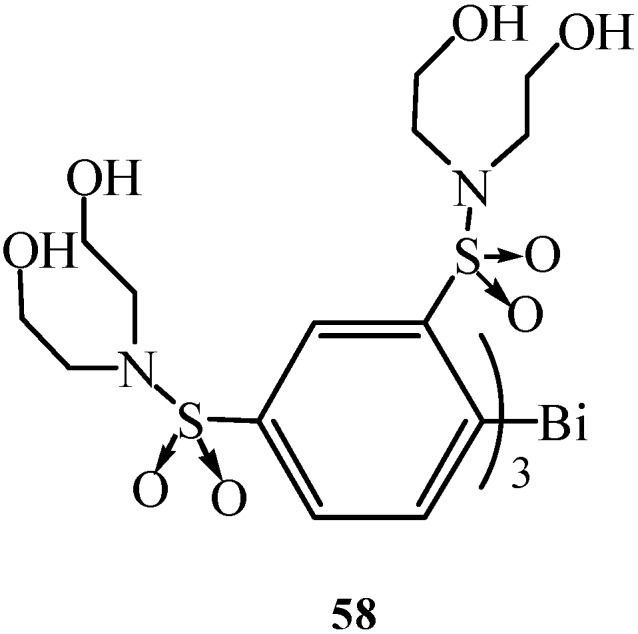
Compound **58** [18].

It was proven that a polybismuth network could be constructed by a simple one-pot polymetalation methodology. In addition, synthesis of thermally and kinetically stabilized oligomeric bismuth compounds bearing more than two bismuth atoms was attractive. Matano *et al.* also synthesized for the first time new water-soluble non-ionic triarylbismuthanes by the combination of sulfonamide function and neutral hydroxy groups [18]. Compound **58** (Figure 2) was claimed to be fairly soluble in water.

**Scheme 23:**
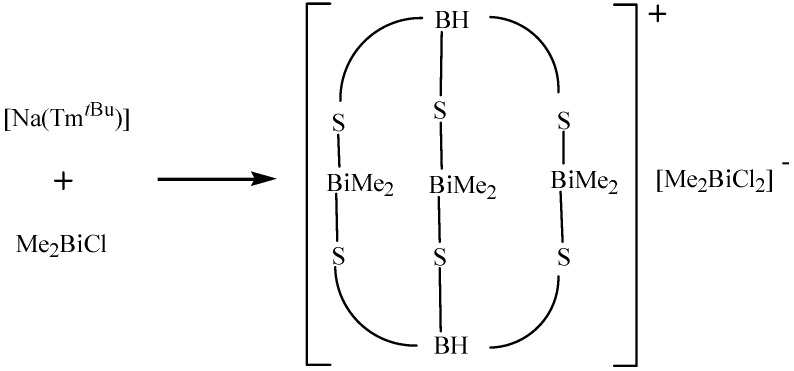
Synthetic reaction for [(Me_2_Bi)_3_(Tm*^t^*^Bu^)_2_]^+^[Me_2_BiCl_2_]^−^ [65].

The first organobismuth rings (RBi)_3_ and (RBi)_4_ (R=(Me_3_Si)_2_CH) were reported by Breunig and co-workers [19]. These compounds were synthesized by reduction of RBiCl_2_ with magnesium filings in THF at –35 ^o^C. The structure of (RBi)_4_ showed a folded four-membered bismuth ring. A cationic trinuclear organobismuth complex with an unprecedented coordination mode in its hydrotris (2-mercaptoimidazolyl)borate ligands was prepared by reaction of [Na(Tm*^t^*^Bu^)] with Me_2_BiCl [65]. Scheme 23 shows the synthetic reaction for [(Me_2_Bi)_3_(Tm*^t^*^Bu^)_2_]^+^[Me_2_BiCl_2_]^−^.

**Scheme 24:**
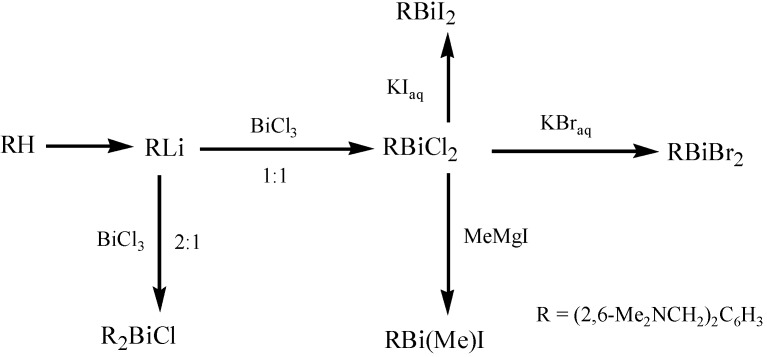
Synthetic route for organobismuth(III) dihalides [26].

Organobismuth(III) dihalides of the type [2,6-(Me_2_NCH_2_)_2_C_6_H_3_]BiX_2_ (X = Cl, Br, I) were the first compounds with a T-shaped CBiX_2_ core [26]. These compounds (Scheme 24) were stabilized by two intramolecular N→Bi interactions *trans* to each other. 

This might be a contributing factor in to their overall distorted square pyramidal (*N*,*C*,*N*)BiX_2_ coordination geometry. Most recently, new hypervalent organobismuth(III) compounds of the types R_3_Bi, R_2_BiCl and RBi*X*_2_ (where R = [2-{*E*(CH_2_–CH_2_)_2_NCH_2_}C_6_H_4_], *E* = O, MeN; *X* = Cl, Br, I) and [2-(Me_2_NCH_2_)C_6_H_4_]BiBr_2_ [66] were prepared with intramolecular N→Bi interactions of different strength. The intramolecular N→Bi interactions resulted in different coordination geometry in these compounds.

**Figure 3:**
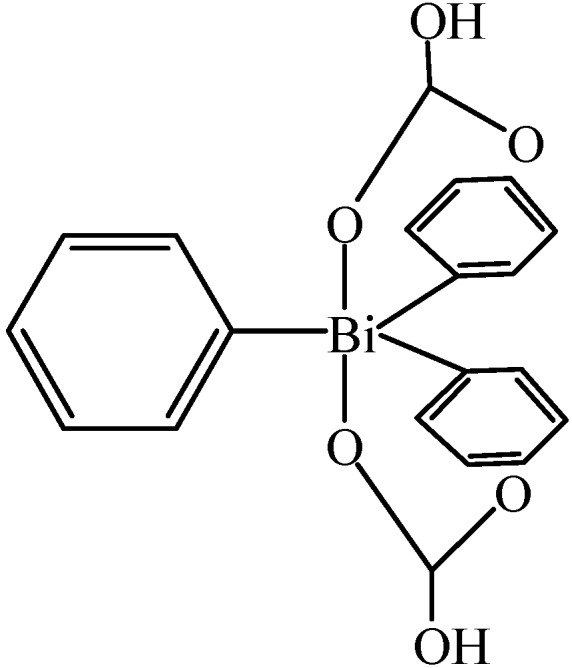
Bismuth(V) complex Bi^V^R_3_(O_2_CR’)_2_ [67].

New bismuth(V) complexes with the formula of Bi^V^R_3_(O_2_CR’)_2_ (Figure 3) have been synthesized by the reaction of BiR_3_Cl_2_ with Ag(O_2_CR’), where R was an aromatic ligand and R’ was a substituent which contained a hydroxyl group [67]. The bismuth atoms in these compounds adopted distorted trigonal-bipyramidal geometries and showed an unusual stereoselectivity towards the chiral ligand R*CO_2_^−^.

A novel binuclear bismuth compound, (Et_2_NCS_2_)_2_(NO_3_)Bi(NO_3_)Bi(S_2_CNEt_2_)_2_(HOCH_3_) was synthesized by Yin *et al*. [68]. As a bismuth derivative of *N*, *N*-diethyldithiocarbamate, this compound was expected to have high biological activity. There are two different bismuth atoms in this compound, one is seven coordinated with distorted pentagonal bipyramidal and the other is eight coordinated with distorted dodecahedron geometry.

## 3. Applications of Organobismuth Reagents in Organic Synthesis

Organobismuth reagents, like many other organometallic reagents, have been well studied and demonstrated to be selective reagents or mild oxidants in organic synthesis [1,69]. For the sake of the goal of environmentally benign reactions, the use of non-toxic reagents has been identified as one of the best procedures in organic synthesis. Thus, a large number of organobismuth mediated reactions had been studied and the fascinating role of organobismuth reagents had been revealed [9,36,38,47]. Many organic reactions such as *C*-, *N*- and *O*-arylation reactions, cross-coupling reactions, polymer reactions, asymmetric synthetic reactions and other special reactions with organobismuth reagents, especially many novel reactions were summarized in the following sections. In the meantime, the reaction pathways or mechanisms of these organobismuth mediated reactions have also been discussed.

### 3.1. Arylation Reactions

Arylation reactions with organobismuth reagents were developed relatively early. Organobismuth reagents allow three major types of arylation reactions: (a) *C*- and *O*-arylation *via* a covalent intermediate; (b) *O*-arylation under neutral conditions; (c) *O*- and *N*-arylation under copper catalysis [9,38]. Previous reviews about various arylation reactions with organobismuth reagents were focused on both the bismuth(V) and bismuth(III) compounds. Both the pentavalent and trivalent states of bismuth were found to display oxidizing power [17], and the selectivity in arylation reactions was dependent on the oxidation state [8]. Besides, the chemoselectivity was strongly dependent upon the choice of the bismuth ligands and the reaction conditions [36].

Arylbismuth compounds could react under extremely mild conditions and afford high yields of the desired product [8,70]. It was said that pentavalent triarylbismuth could be widely used under mild conditions and excellent yields of products could be achieved [71,72]. The main problem was that commonly only one aryl group of the triarylbismuth reagents could be eventually transferred [36]. Moreover, limitations in the method of preparing these reagents limited the functionality on one of the two organic groups in the coupling event [8]. In retrospect, the reported examples of pentavalent triarylbismuth derivatives showed much of researchers’ efforts in exploring the reactivity of organo-bismuth reagents.

**Scheme 25:**
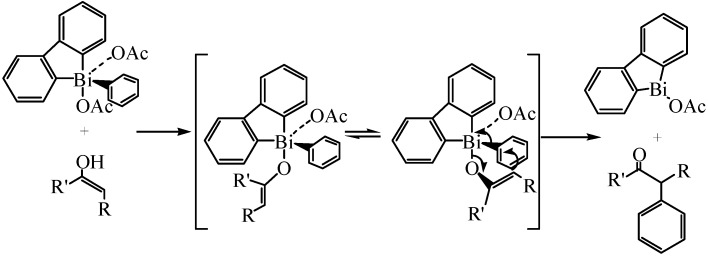
Mechanism of the ligand coupling step [36].

Pentavalent biphenyl-2,2’-ylenephenylbismuth diacetate [36] was reported to react with nucleophiles under basic conditions providing modest to good (79%) yields of the corresponding *C*-phenylated substrates, while this compound reacted with hydroxy or amino groups providing the products of *O*- or *N*-phenylation under copper catalysis. For biphenyl-2,2’-ylenephenylbismuth diacetate, two aryl groups were included in a heterocyclic bismuth substructure to make a stabilized form of these two ligands. Hence, the loss of two ligands would be avoided and result in the selective transfer of the third aryl group. Above results showed that insertion of two phenyl groups of triphenylbismuth into a cyclic system allowed the selective transfer of only the free phenyl group (Scheme 25) both in base-catalysed *C*-phenylation and in the copper-catalysed *O*- and *N*-phenylation reactions. However, insertion of the bismuth atom into the five membered ring led to a decrease of the reaction rates.

BiPh_3_Cl_2_ is one of the well-known triarylbismuth(V) reagents capable of effecting many arylation reactions [73]. Koech *et al.* [74] reported a special method for the regiospecific α-arylation of enones and enals by using BiPh_3_Cl_2_ reagents under nucleophilic catalysis conditions. The reactions which were described in Scheme 25 afforded the corresponding α-aryl enones and α-aryl enals in good to excellent yield (93%). The use of tributylphosphine was a complement to the related Pd-catalyzed methods for enolate arylation in several respects.

**Scheme 26:**
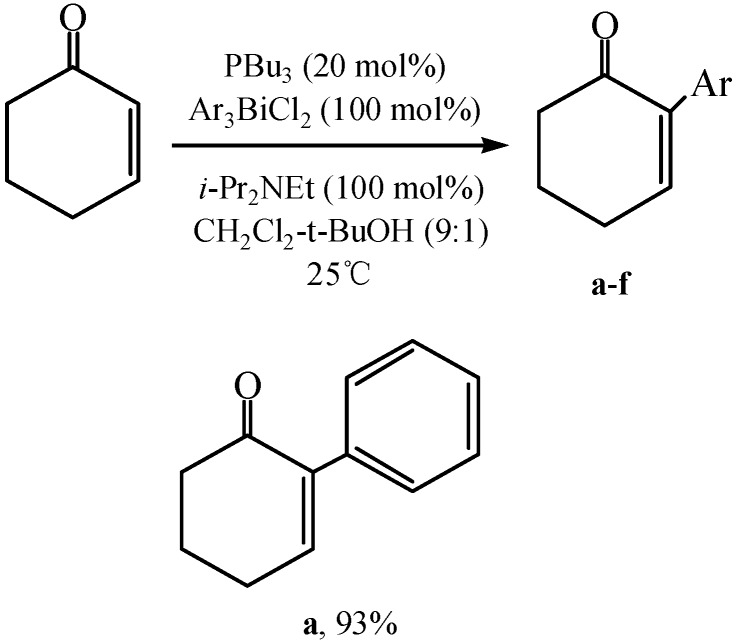
Phosphine catalyzed arylation of cyclohexenone by using BiPh_3_Cl_2_ reagents [74].

Ph_3_Bi(OAc)_2_ is another type of common triarylbismuth(V) reagent that has found application in *N*-arylations with aminoindazoles [75]. Mono-*O*-phenylation of diols with Ph_3_Bi(OAc)_2_ afforded the monophenyl ether in good yield [76]. Cu(II)-catalyzed *O*-phenylation of functionalized tertiary alcohols such as α-hydroxycarbonyl compounds with Ph_3_Bi(OAc)_2_ or tetraphenylbismuth compounds (Ph_4_BiF) provided *tert*-alkyl phenyl ethers in good yields [77]. With regard to tetraphenylbismuth compounds (Ph_4_BiX), these were representative reagents for the phenylation of various organic molecules [47]. The first report on fluorotetraphenylbismuth (Ph_4_BiF) which was published by Ooi *et al.* [47] demonstrated its applications for the efficient α-phenylation of ketones and esters. Based on the utilization of the inherent basicity of the fluorine atom, Ph_4_BiF was thermally stable and maintained its amphiphilic property of possessing both nucleophilic and electrophilic moieties within a molecule. Therefore, the problem that tetraphenylbismuth(V) compounds with a strongly coordinating (highly nucleophilic) anion decomposed rapidly could be solved.

**Scheme 27:**
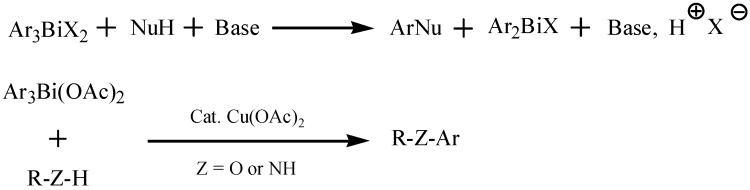
Arylation reactions under basic conditions andcopper-catalyzed arylation reactions [9].

Electronic factors were not the major limiting feature for the usefulness of the bismuth-mediated arylation reactions. Finet *et al.* recently investigated the reactivity of a series of tris(polymethoxyphenyl)-bismuth derivatives in typical examples of *C*-, *N*- and *O*-arylation reactions [9]. The results indicated that bismuth compounds which bore electron-rich aryl groups were valuable and attractive reagents for the transfer of aryl fragments with electron-donating substituents in the arylation reactions (Scheme 27), affording good to high yields of the corresponding *C*-, *N*- and *O*-arylation products.

**Scheme 28:**
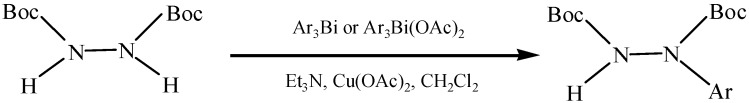
Arylation of diversely substituted hydrazines using Ar_3_Bi or Ar_3_Bi(OAc)_2_ [79].

Arylation of hydrazines is attractive since hydrazine derivatives play important roles in the agrochemical and dye-stuff industries and a number of pharmaceuticals [78,79]. In order to design triprotected reagents for the stepwise synthesis of hydrazine derivatives, Tšubrik *et al.* had utilized pentavalent organobismuth reagents in the selective arylation of disubstituted hydrazines [78]. Compounds of the type R^1^NHNHCOR^2^ could be selectively arylated under very mild conditions by using Ar_3_Bi(OAc)_2_ and provide the product R^1^ArNNHCOR^2^ in good yields (74–95%). Specifically, some of the arylation reactions could be accomplished even without a copper catalyst. In addition, pentavalent organobismuth reagents [Ar_3_Bi(OAc)_2_ or Ph_3_Bi(OAc)_2_] were compared with trivalent ones (Ar_3_Bi or Ph_3_Bi) in arylation of diversely substituted hydrazines (Scheme 28) [79]. It was found that tri- and pentavalent organobismuth reagents could complement each other with respect to arylation on nitrogen. Specifically speaking, the results showed apparent advantages of pentavalent over trivalent reagents in the case of mono- and disubstituted hydrazines. In contrast, trisubstituted hydrazines were more efficiently substituted by trivalent reagents.

**Scheme 29:**
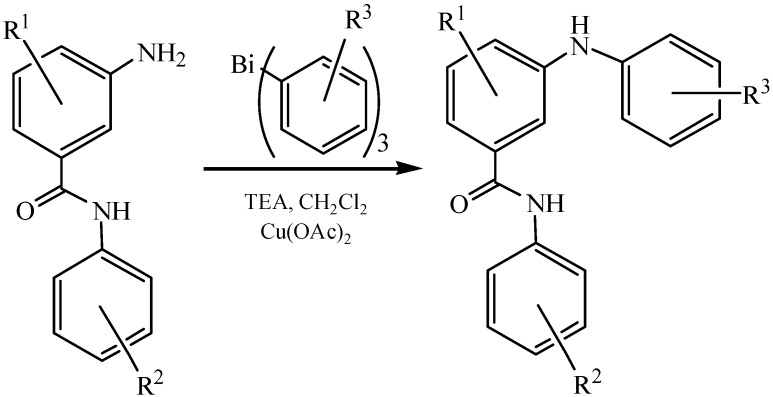
Selective *N*-arylation of 3-aminobenzanilides [80].

Trivalent organobismuth reagents such as triarylbismuthanes (Ar_3_Bi) are available, easily handled reagents for the selective *N*-arylation of amino groups in functionalized aminobenzanilides under mild conditions (Scheme 29) [80]. The diarylamine products were obtained selectively in good yields (46%–94%). The drawback that only one of the aryl groups of the bismuth reagent was transferred to the substrate was unavoidable in this method.

**Scheme 30:**
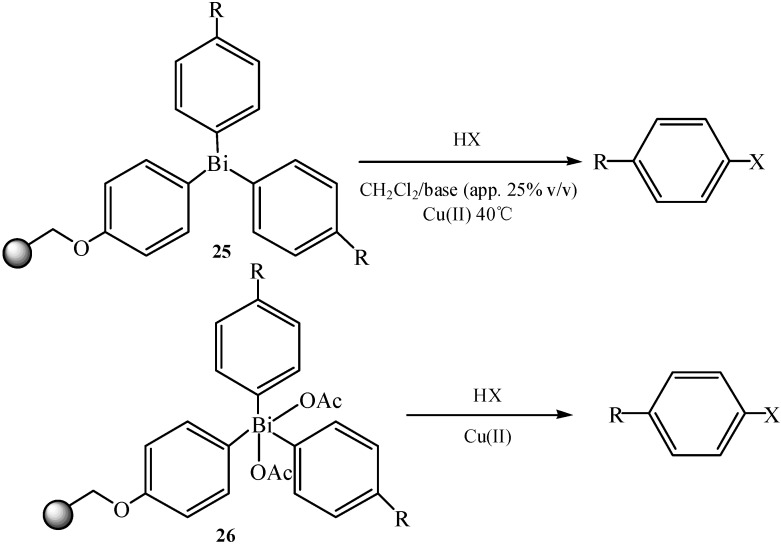
Cleavage from resins **25** and **26** [50].

Special triarylbismuthanes **25** and triarylbismuth diacetates **26** were prepared by taking advantage of solid-phase chemistry [50]. The as-prepared resin-bound bismuths were utilized as resin-bound arylation reagents in *O*-, *N*- and *C*-arylations. The obtained products were of high diversity and were obtained in moderate to good yields. The multidirectional linker system allowed the synthesis of different scaffolds by the introduction of a wide range of diverse fragments in the final cleavage step (Scheme 30). Thus, resin-bound bismuth as a multidirectional linker system showed this advantage and also compensated for the lack of selectivity by the cleavage of aryl groups from the bismuth.

### 3.2. Cross-coupling Reactions

The metal-catalyzed cross-coupling reaction of organic electrophilic reagents with organometallic reagents is one of the most important synthetic methods for C-C bond formations, in particular for those containing sp^2^- or sp-C atoms [81,82,83]. Though many organometallic compounds such as organo- tin, organoboron and organozinc compounds had been successfully utilized in cross-coupling reactions, the search for new nucleophilic reagents that could serve in sub-stoichiometric amounts with respect to electrophilic partners was still underway [1]. It was gratifying that organobismuth compounds were demonstrated to be useful reagents for the cross-coupling reaction with various electrophilic reagents (Table 2).

Organobismuth compounds such as triarylbismuth reagents are referred to as atom-efficient organometallic coupling partners for C–C bond formations. The reason was that triarylbismuth reagents could react with more than 1 equivalent of electrophilic coupling reagents in order to reduce the organometallic loadings for industrial scale preparations [87]. Thus, triarylbismuth reagents with three aryl groups on bismuth allowed the development of atom-economic coupling reactions. It was reported that moderate to good yields was achieved in the cross-coupling reaction of triarylbismuth compounds (BiAr_3_) with aryl iodides under mild conditions [88].

**Scheme 31:**
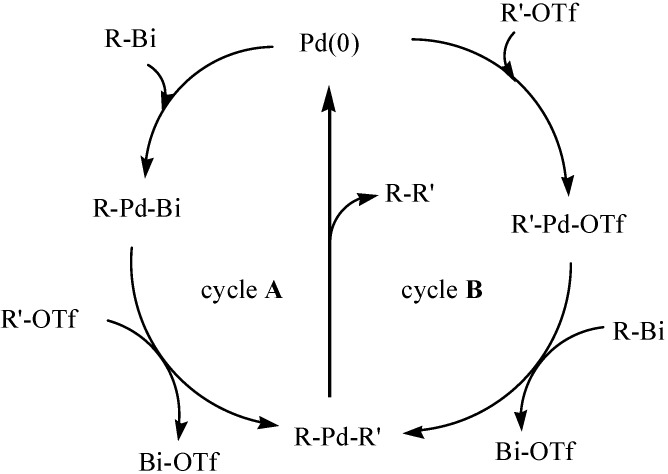
Two possible catalytic cycles [84].

It could be seen from Table 2 that organobismuth dialkoxides (Scheme 31) showed high reactivity in the cross-coupling reactions with aryl and vinyl triflates as well as with aryl bromides and iodides [84,81]. The researchers suggested the two possible catalytic cycles—Cycle A and cycle B—shown in Scheme 31. It was suggested that cycle A ocurred because of the weak Bi–C bond, and it was envisioned to readily add to Pd(0) species. Cycle B is similar to those which are generally accepted for the cross-coupling reactions of organotin and organoboron compounds.

**Table 2:** Cross-coupling reactions of organobismuth reagents with electrophilic partners.

Reagent	Reaction	Catalyst	Yield, % (highest)	References
Organobismuth dialkoxides	Cross-coupling with aryl and vinyl triflates	Pd(PPh_3_)_4_	99	[84]
Organobismuth dialkoxides	Cross-coupling with aryl bromides and iodides	Pd(PPh_3_)_4_	99	[81]
Triarylbismuths	Cross-coupling with aryl halides and triflates	PdCl_2_/PPh_3_	96	[1]
Triarylbismuths	Cross-coupling with α,β-unsaturated acyl chlorides	PdCl_2_/PPh_3_	91	[10]
Triarylbismuths	Cross-coupling with allylic carbonates	PdCl_2_(PPh_3_)_2_	90	[85]
Resin-boundtriarylbismuthanes	Suzuki cross-coupling with aryl boronic acids	Pd_2_dba_3_ andtri- *tert*-butylphosphane	83	[51]
Triarylbismuths	Cross-coupling with aryl bromides or iodides	polystyrene-supported Pd^II^	94	[82]
Triarylbismuths	Multi-coupling with vinylic iodides	PdCl_2_(PPh_3_)_2_	85	[83]
Triarylbismuths	Domino coupling with 1,1-dibromo-1-alkenes	Pd(PPh_3_)_4_	88	[86]
Triarylbismuths	Multi-coupling with bromide and chloride derivatives of Baylis–Hillman adducts	Pd_2_dba_3_	91	[87]

**Scheme 32:**
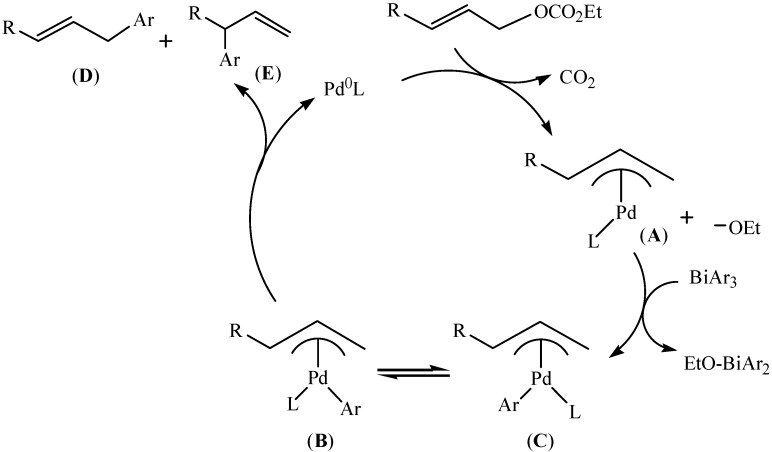
Proposed catalytic cycle [85].

**Scheme 33:**
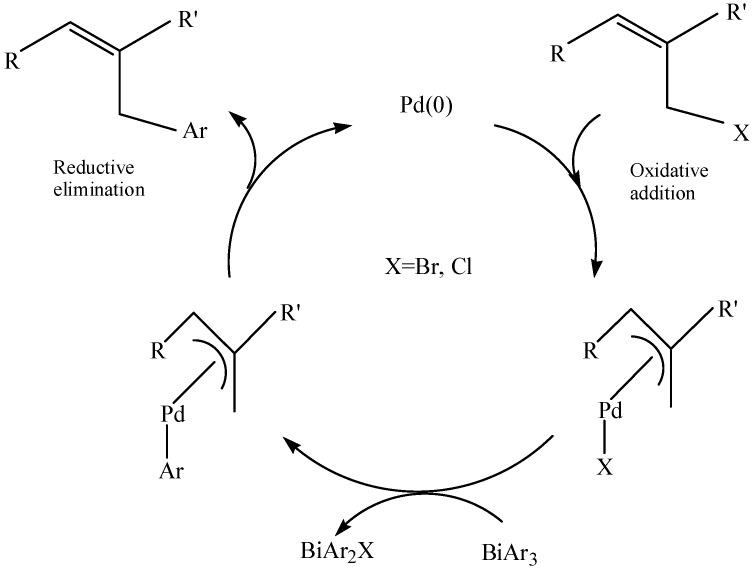
Proposed mechanistic cycle [87].

The tentative catalytic cycle (Scheme 32) which was proposed in reference [85] was expected to go through a π-allyl-palladium alkoxide intermediate (A). In this cycle, triarylbismuths were multi-coupling organometallic nucleophiles with allylic carbonates for three C–C couplings. Besides, the product (E) was an unavoidable minor product by the homo-coupling of triarylbismuths [86]. Another mechanistic cycle (Scheme 33) which was proposed in reference [87] also referred to the formation of π-allyl palladium intermediate.

**Scheme 34:**
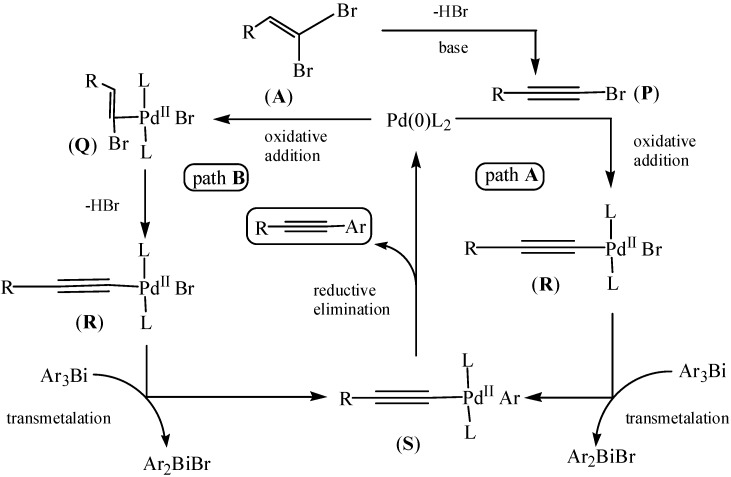
Proposed mechanism [86].

The domino couplings of triarylbismuths with three equivalent of 1,1-dibromo-1-alkenes were very fast, affording high yields of alkynes in a short reaction time [86]. As depicted in Scheme 34, the proposed cycles indicated two domino coupling cycles, path A and path B. Both cycles involved an alkyne-Pd intermediate (**R**). It was worthwhile to mention that the Ar_2_BiBr or ArBiBr_2_ species could participate in subsequent catalytic cycles during transmetalation.

### 3.3. Asymmetric Synthesis

**Scheme 35:**
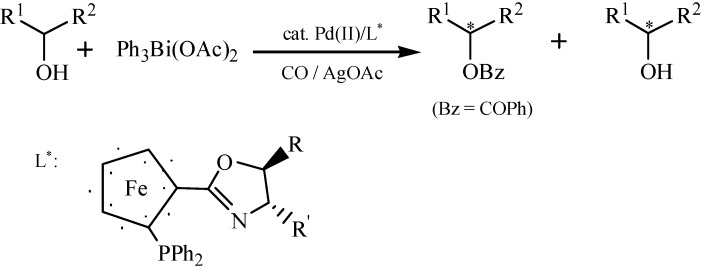
Kinetic resolution of secondary alcohols [22].

Asymmetric synthesis is an important and useful tool in modern synthetic organic chemistry [89] and various organometallic reagents are involved in many asymmetric reactions [89,90]. Organo- bismuth reagents could act as basic reagents because of a pair of electrons in their highest s orbitals [91]. However, applications of organobismuths in asymmetric synthesis were relatively scarce till now [22]. Miyake *et al.* opened up new realms for organobismuth investigation [22,92]. Pd(II)-catalyzed kinetic resolution of racemic secondary alcohols *via* their enantioselective benzoylation by using CO and organobismuth(V) compound had been developed (Scheme 35) [22]. It was mentioned that although satisfactory enantioselectivity was not obtained (up to 48% *ee*), this reaction system seemed to be interesting from the viewpoint of both asymmetric synthesis and organobismuth chemistry.

**Scheme 36:**
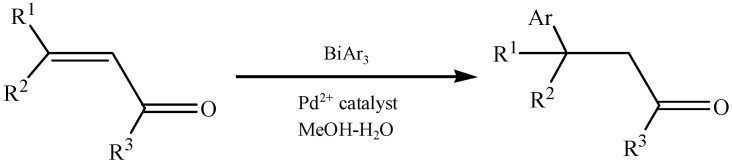
Asymmetric addition of Ar_3_Bi to enones [90].

Nishikata *et al.* have reported an asymmetric 1,4-addition of triarylbismuths to cyclic and acyclic enones in aqueous methanol catalyzed by a chiral phosphine-dicationic palladium(II) complex [90]. The reaction system (Scheme 36) provided the products optically active β-arylketones of up to 95% *ee*.

**Scheme 37:**
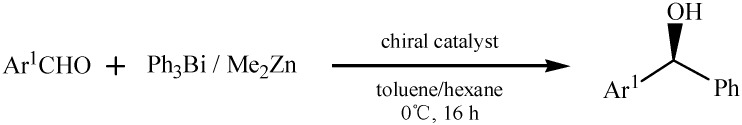
An enantioselective phenyl transfer reaction by using a mixed Ph_3_Bi/Me_2_Zn reagent [89].

Another highly enantioselective reaction (Scheme 37) was carried out by Sato *et al.* [89]. The aryl transfer reactions to aromatic aldehydes by using mixed triarylbismuthane and dimethylzinc reagents provided diarylmethanols in good yield (up to 97%). However, the active species of the mixed Ph_3_Bi/Me_2_Zn reagent is still unknown. It was difficult to know whether transmetallation products were formed or not. Otherwise a metal exchange reaction might happen between dialklyzinc and organo- bismuth reagents.

### 3.4. Other Special Reactions

**Scheme 38:**
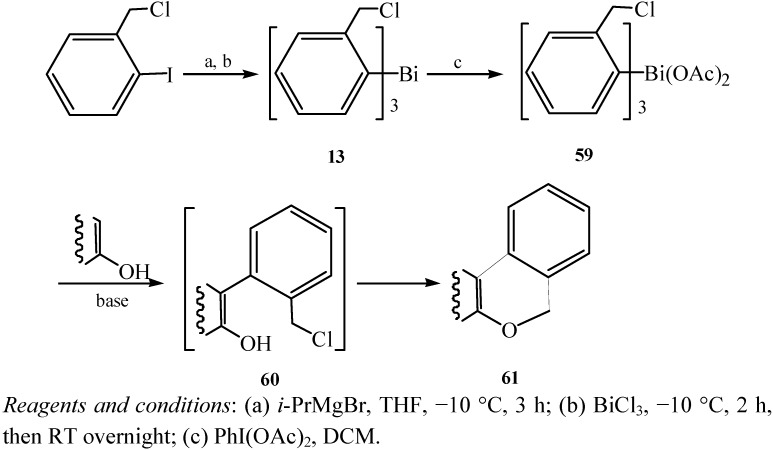
Synthetic route for dibenzo[*b*,*d*]pyran derivatives [43].

A new synthetic route to dibenzo[*b*,*d*]pyran derivatives by using organobismuth-mediated one-pot reaction was developed [43]. As depicted in Scheme 38, triarylbismuth diacetate (**59**) resulted from the oxidation of Bi(III) derivative **13** and subsequent *ortho*-arylation reaction afforded product **60**. Finally, a spontaneous intramolecular cyclisation led to the formation of compound **61**. Though the yields of dibenzo[*b*,*d*]pyran derivatives were not so satisfactory compared with those possible using an organolead reagent, the environmentally friendly nature of the organobismuth variant should be noted.

**Figure 4:**
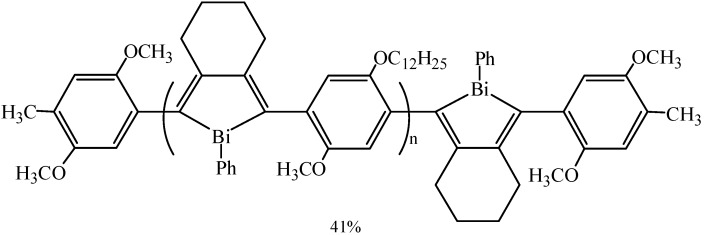
Bismuth-containing polymer [93].

The synthesis of bismuth-containing polymers had been rarely reported, not to mention the construction of conjugated polymers containing the bismole skeleton in the main chain. Morisaki *et al.* [93] first synthesized a conjugated polymer (Figure 4) containing a bismuth atom in the conjugated main chain by incorporating the bismuth atom into the cyclopentadiene skeleton (bismole skeleton). The as-prepared polymer was generated in 41% isolated yield and exhibited moderate bluish photoluminescence in solution. In addition, Pu *et al*. [94] have mentioned that some recently developed organobismuth compounds were effective mediators for the living radical polymerization of most *N*-vinylamides.

Maoecrystal V was a Traditional Chinese Medicine component with an unusual structure. The group of Krawczuk [95] recently described an enantioselective synthesis of the maoecrystal V carbon skeleton. Remarkably, one of the key transformations was the arylation of a 1,3-dicarbonyl compound with a triarylbismuth(V) dichloride species (Ar_3_BiCl_2_).

**Scheme 39:**
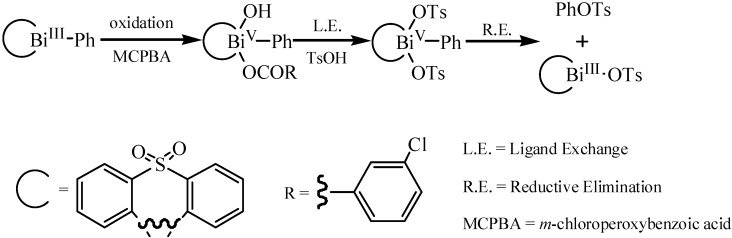
A plausible mechanism for the formation of the phenyl tosylate [69].

The unique character of valency change from Bi^V^ to Bi^III^ allows pentavalent organobismuths to be effective reagents for *O*-phenylation of tertiary alcohols [96] and oxidative coupling reactions of carbonyl compounds [97], and also for synthesis of aryl tosylates [69]. In addition, two ligands on the pentavalent bismuth were coupled accompanied with reductive elimination to simultaneously generate trivalent bismuth. A novel ligand coupling reaction between an aryl group and a tosyloxy group on the pentavalent bismuth established by Sakurai *et al.* [69] afforded aryl tosylates in good to high yields. A plausible mechanism for the formation of the phenyl tosylate is shown in Scheme 39. Specially, the pentavalent bismuth ditosylates which were used in the reaction were heterocyclic bismuth compounds. Heterocyclic bismuth compounds were reported to be quite different from the noncyclic ones [98].

## 4. Applications of Organobismuth Catalysts in Organic Synthesis

A diversity of inorganic bismuth(III) compounds such as Bi(OTf)_3_, Bi(TFA)_3_, BiCl_3_, BiBr_3_, Bi(NO_3_)_3_, *etc.* had been previously reported as effective catalysts in various organic reactions due to their Lewis acid character and the environmentally friendly nature of bismuth compounds [12,60,99,100,101,102,103]. However, the unstable nature of Bi–C bonds of bismuth compounds has resulted in rare reports on the use of organobismuth compounds as catalysts [59]. In order to develop suitable organo- bismuth compounds for catalytic utilization, several advanced research works have reported about organobismuth catalysts. Thus, these works might offer some enlightenment to the development of organobismuth catalyzed reactions.

### 4.1. Cationic Organobismuth Complexes

Cationic organometallic complexes of transition and main group metals such as Y, La, Sm, Ni, Pb, *et al.* [104] have been widely used as catalysts for polymerization and organic synthesis. However, for cationic organobismuth complexes, there were not examples of applications in catalysis until the study reported by Bao *et al.* [28]. Cationic 5,6,7,12-tetrahydrodibenz[*c*,*f*][1,5]azabismocine with a weakly coordinating borate anion and its coordination complexes with some neutral donor molecules were synthesized. The preliminary evaluation of the catalytic performance of the cationic complex ([*^t^*BuN(CH_2_C_6_H_4_)_2_Bi]^+^[B(C_6_F_5_)_4_]^−^) showed that this complex worked as a Lewis acid catalyst for allylation reaction of aldehydes with allylbismuth species and the Mukaiyama aldol reaction.

This breakthrough showed the potential of the use of cationic organobismuth complexes for catalysis and also highlighted the synthetic possibility of stable organobismuth compounds by adoption of stable cyclic frameworks [53,54]. To achieve this purpose, recent studies have brought forth new ideas in cationic organobismuth catalyzed reactions.

Air-stable cationic organobismuth(III) compound [S(CH_2_C_6_H_4_)_2_Bi(OH_2_)]^+^[ClO_4_]^−^ with both Lewis acidity and basicity efficiently catalyzed the direct diastereoselective Mannich reaction in various solvents, including water [58]. There was difficulty in carrying out Mannich reactions over bifunctional catalysts which were composed of a Lewis acid and Lewis base. Moreover, the rarely reported examples with low yield, poor diastereoselectivity or environmentally unfriendly disadvantage were not enough. This cationic organobismuth(III) compound exhibited high activity, diastereoselectivity, stability and reusability compared with other bismuth catalysts, and its *anti*-selectivity was almost independent of the solvents. Especially, high efficiency (yield 98%) and stereoselectivity (*syn*:*anti* = 5:95) were obtained using water as the solvent. Thus, the as-prepared cationic organobismuth(III) compound highlighted its catalytic application in organic synthesis.

**Scheme 40:**
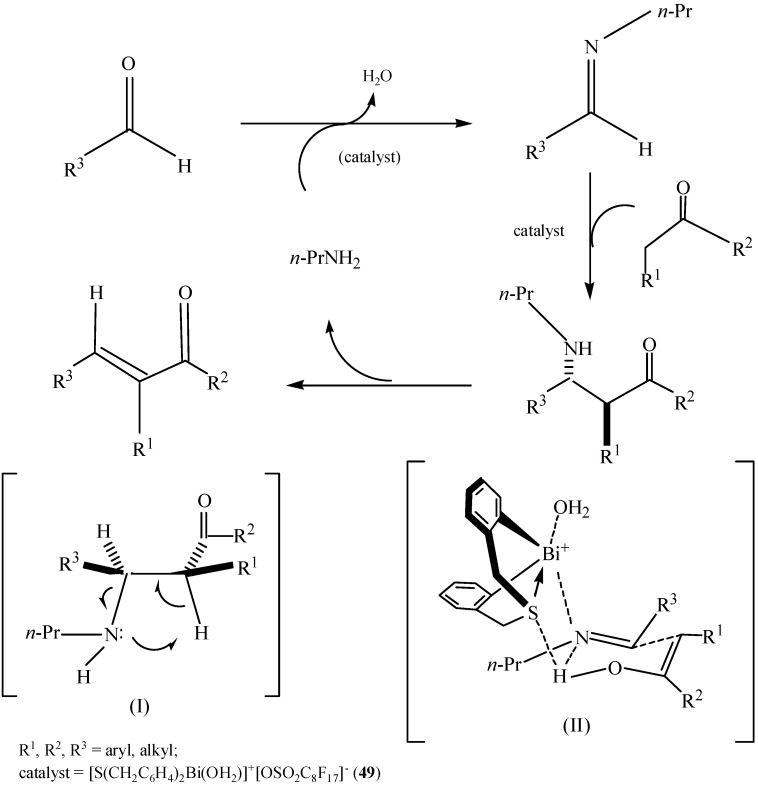
A plausible catalytic cycle for the crossed-condensation reaction of ketones and aldehydes [59].

Another cationic organobismuth perfluorooctanesulfonate [S(CH_2_C_6_H_4_)_2_Bi(OH_2_)]^+^[OSO_2_C_8_F_17_]^−^ (**49**) also showed Lewis acidity for the stereoselective synthesis of (*E*)-α,β-unsaturated ketones in water [59]. Experiments were carried out for one-pot synthesis of (*E*)-α,β-unsaturated ketones through the direct crossed condensation of aldehydes and ketones in various solvents, including water, providing the target products in good yields (up to 95%) without multistep transformations and product separation. High diastereoselectivity (*anti*/*syn* > 99/1) and mild conditions made the new synthetic process superior to the common processes. As shown in Scheme 40, the reactions probably took place through a Mannich-type mechanism. The sulfur atom in compound **49** could act as a weak Lewis base, and the high stereoselectivity of **49** might have some connection with that Lewis base. Another green synthesis of (*E*)-α,β-unsaturated ketones was catalyzed by a new bifunctional complex [S(CH_2_C_6_H_4_)_2_Bi(OH_2_)]^+^[BF_4_]^−^ in a readily separated catalyst system [105]. This catalyst system developed by Qiu *et al*. not only afforded the products with high catalytic efficiency, but also featured facile separation and reusability of the catalyst.

### 4.2. Air-Stable Organobismuth Compounds

**Scheme 41:**
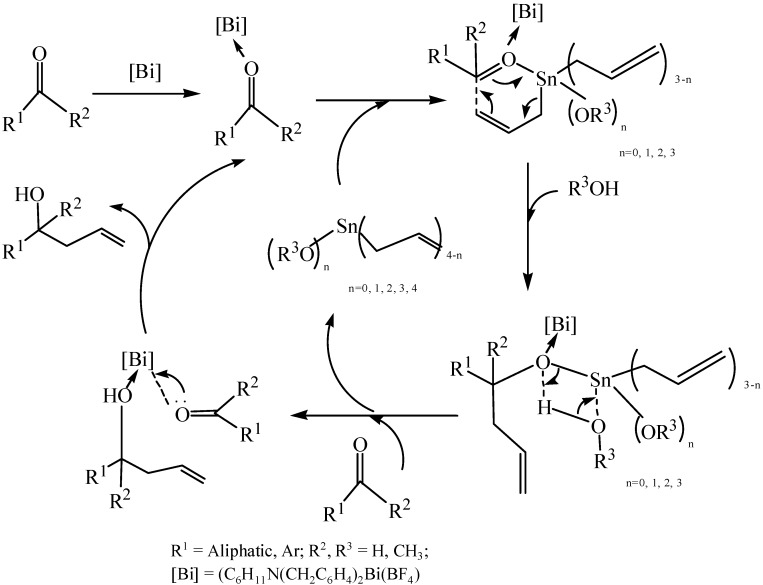
Possible catalytic mechanism for the allylation of aldehydes catalyzed by C_6_H_11_N(CH_2_C_6_H_4_)_2_BiBF_4_ [60].

In order to obtain organometallic compounds that had potential uses in catalysis, as previously stated, a stable metal-carbon bond was required. On the other hand, proper counteranions were needed to stabilize the structures and to improve the catalytic activity. Stable cyclic frameworks could be adopted in the synthesis of organobismuth derivatives to get stable organobismuth compounds [28]. In addition, acidity and stability of organometallic (e.g., Sn, Ti, Zr, Hf) complexes could be enhanced by the incorporation of long-chain perfluoroalkylsulfonate and perfluoroarylsulfonate groups [24]. Referring to a preceding part of the text (Section 2.1), Zhang and co-workers have synthesized a series of air-stable organobismuth compounds which were proven to be excellent catalysts in organic reactions such as allylation reactions and Mannich reactions. The allylations of aldehydes and ketones with tetraallyltin were conducted by a Lewis acid catalyst which was identified as the hypervalent organobismuth(III) tetrafluoroborate C_6_H_11_N(CH_2_C_6_H_4_)_2_BiBF_4_ [60]. Reactions occurred smoothly in various solvents such as MeOH, C_2_H_5_OH, CH_3_CN, *etc.* and the presence of electron-withdrawing or electron-donating groups on the aldehydes had little effect on the reaction. The expected homoallylic alcohols were formed with excellent chemoselectivity and yields as high as 97%. Researchers have postulated the mechanism of the allylation reaction over C_6_H_11_N(CH_2_C_6_H_4_)_2_BiBF_4_ in aqueous methanol solution (Scheme 41). C_6_H_11_N(CH_2_C_6_H_4_)_2_BiBF_4_ was regenerated by the cleavage of the coordinate bond, and was ready for the next catalytic cycle. The bismuth center of this compound acted as a Lewis acid site and the electron-withdrawing group (BF_4_) was strong.

**Scheme 42:**
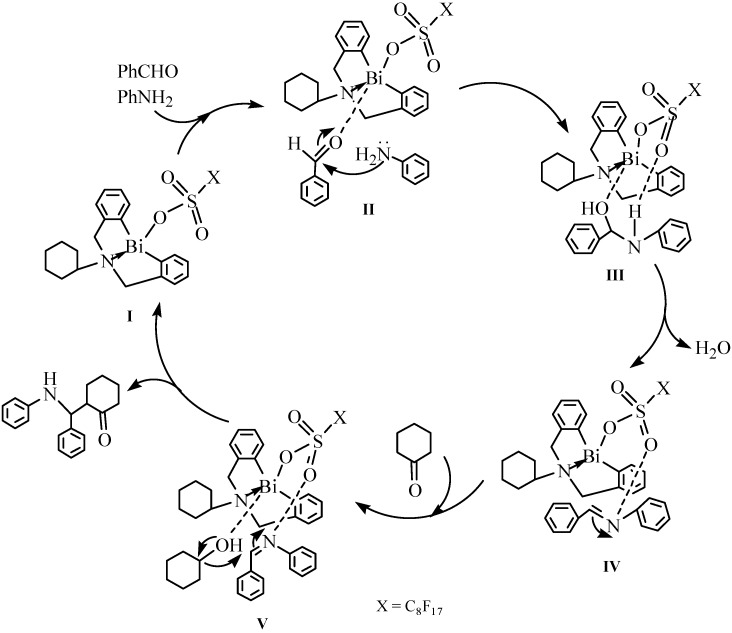
Proposed mechanism on the direct Mannich reaction catalyzed by organobismuth perfluorooctanesulfonate [24].

The one-pot three-component Mannich reactions of aldehydes, amines and ketones in water catalyzed by organobismuth(III) perfluorooctanesulfonate [C_6_H_11_N(CH_2_C_6_H_4_)_2_Bi(OSO_2_C_8_F_17_)] [24] were proposed to ocurr according to the mechanism described in Scheme 42. It can be seen from this Scheme that the carbonyl oxygen atom of benzaldehyde coordinated with the bismuth atom of the catalyst (I) and was thus activated. As a result, an intermediate (II) was formed. Thus, the bismuth atom of the catalyst played an important role during the catalytic procedure.

### 4.3. Carbon Dioxide Fixation by Organobismuths

**Scheme 43:**
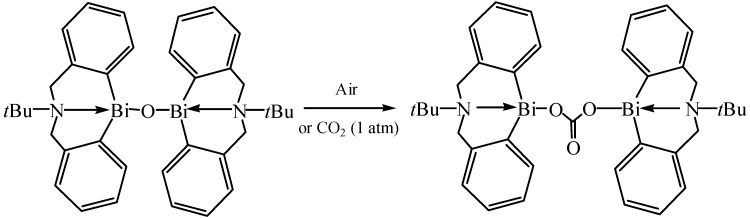
Reaction of bismuth oxide with CO_2_ [107].

**Figure 5:**
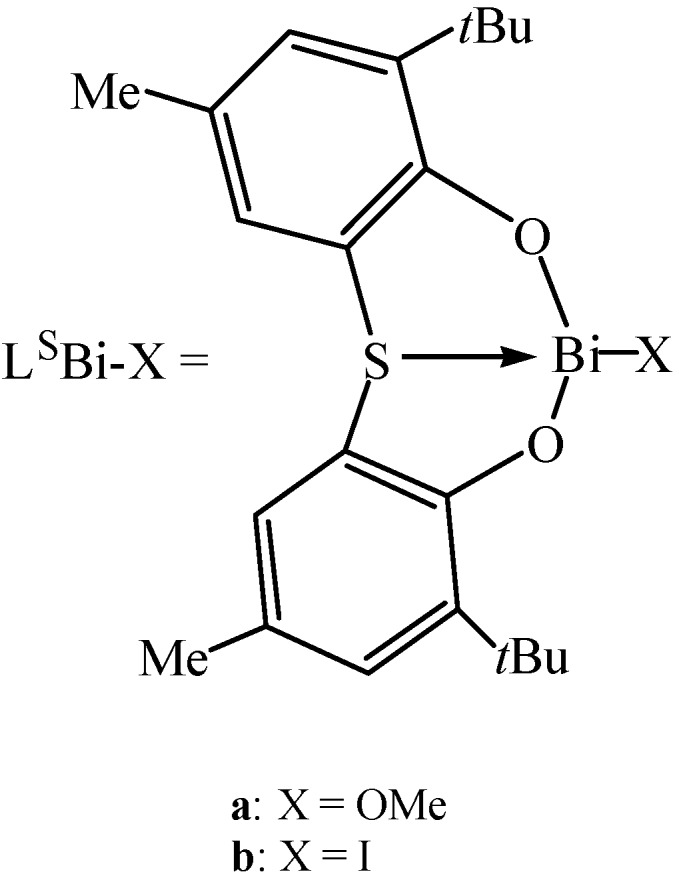
New bismuth compounds [108].

Chemical fixation of CO_2_ is an attractive field and many metal compounds can be used for CO_2_ fixation [106]. Yin and co-workers have reported the use of organobismuth oxides, hydroxides, and alkoxides for CO_2_ conversion into useful chemicals as well as for CO_2_ capture and separation [107]. As shown in Scheme 43, the CO_2_ fixation by bismuth oxide was irreversible at room temperature. These compounds were synthesized with the 5,6,7,12-tetrahydrodibenz[*c*,*f*][1,5]azabismocine framework. Other two new bismuth compounds which possess a sulfur-bridged bis(phenolato)ligand (Figure 5) were used for solvent-free synthesis of propylene carbonate from CO_2_ and propylene oxide by Yin and co-workers [108]. The new compounds were thermally stable and showed high catalytic activity for the coupling of CO_2_ with propylene oxide.

## 5. Conclusions

In conclusion, as an environment-friendly class of organometallic compound, organobismuth compounds participated in new, simple, efficient and ‘green’ protocols for organic synthesis, catalytic processes and even synthesis of materials. During the last two decades, interest in organobismuth compounds and the applications of organobismuth compounds had already promoted their development with a great step forward, and some remarkable progress had been achieved. Nonetheless, there were still problems hindering more widespread applications of organobismuths. As described in the present review article, though arylation with organobismuths afforded high yields in the products, the aryl groups of the bismuth reagent could not be fully transferred and the synthesis of new reagents seemed unsatisfactory. Application of organobismuths in C–C bond formations and asymmetric synthesis remain a tough topic for future studies. Besides, many reaction mechanisms for some special reactions with organobismuths are still not clarified. Thus, the search for new and useful reactions with bismuth compounds, especially organobismuths, is expected to be time consuming but full of interest. Overall, the future is bright for these organobismuth compounds, and we look forward to the great progress in this area.
